# Cyclin K condensates bridge CDK12 to phosphorylate and drive oncogenic YAP activation in hepatocellular carcinoma

**DOI:** 10.1126/sciadv.aec6492

**Published:** 2026-07-03

**Authors:** Yang Sun, Yu Zhang, Weikang Yan, Jinlin Duan, Ke Qiao, Guoquan Yan, Junyan Xue, Jie Wang, Ming Zhan, Qiwei Li, Hongcheng Wang, Yonglong Zhang

**Affiliations:** ^1^ Laboratory of Targeted Therapy and Precision Medicine, Department of Clinical Laboratory, Shanghai Jiao Tong University Affiliated Sixth People’s Hospital, Shanghai, China.; ^2^ Department of Biliary-Pancreatic Surgery, Renji Hospital, School of Medicine, Shanghai Jiao Tong University, Shanghai, China.; ^3^ Department of Pathology, Tongren Hospital Shanghai Jiaotong University School of Medicine, Shanghai, China.; ^4^ Shanghai Key Laboratory of Metabolic Remodeling and Health, Institute of Metabolism and Integrative Biology, Fudan University, Shanghai, China.; ^5^ Institute of Biomedical Sciences, Shanghai Medical College, Fudan University, Shanghai, China.; ^6^ Department of Systems Biology, Beckman Research Institute, City of Hope, Monrovia, CA 91016, USA.; ^7^ Department of General Surgery, Shanghai Sixth People’s Hospital Affiliated to Shanghai Jiao Tong University School of Medicine, Shanghai, China.

## Abstract

Targeting transcriptional condensates is an emerging paradigm for cancer therapy. A key player is the transcriptional coactivator YAP (Yes-associated protein), which drives tumor-specific programs that fuel tumor progression and therapeutic resistance. Cyclin K, partnered with cyclin-dependent kinases (CDKs) CDK12/CDK13, is essential for transcription elongation, but its role in specific oncogenic programs was unclear. Here, we identify Cyclin K as an essential vulnerability across multiple cancer types. The CDK12/Cyclin K complex binds YAP via Cyclin K and forms a regulatory condensate to bridge YAP phosphorylation by CDK12. Such a phosphorylation at threonine-398 impedes YAP inhibition by its canonical LATS kinases, stabilizes YAP, and enables its further condensation with TEAD4 to stimulate YAP oncogenic activity. Coexpression of CDK12/Cyclin K and YAP predicts sensitivity to Cyclin K inhibitors in hepatocellular carcinoma cells and patient-derived xenografts. Thus, we define CDK12/Cyclin K as a critical regulator of YAP-driven transcriptional addiction and a biomarker for patient stratification who mostly benefit from therapies targeting the CDK12/Cyclin K–YAP axis.

## Teaser

Protein condensation is a targetable vulnerability for treating a subset of liver tumors.

## INTRODUCTION

The selective mRNA transcription is a fundamental biological process and is tightly controlled by the transcriptional machinery, including RNA polymerase II (Pol II), transcription factors and associated cofactors, and regulatory elements within the DNA (*1*, *2*). Transcription is often dysregulated in diseases, including tumors (*1*, *3*). Tumor initiates from genetic alterations that drive dysregulated transcriptional programs and establish specific transcriptional addictions to sustain numerous oncogenic phenotypes. It is thus well documented as the most fundamental feature of cancer (*3*–*5*), including hepatocellular carcinoma (HCC). Similar to cell cycle regulation, Pol II–dependent gene transcription is controlled by the concerted action of multiple transcriptional CDK-cyclin pairs, including CDK7, 8, 9, 12, and 13 and their cognate cyclins (*3*, *6*, *7*). The catalytic activity of the Pol II complex throughout transcription cycles is tightly linked to the dynamic phosphorylation of the heptad (YSPTSPS) amino acid repeats within the C-terminal domain (CTD) of the RPB1 subunit (*8*, *9*). Phosphorylation of serine at position 5 (S5) of the CTD is required for proper transcriptional initiation from gene promoters, whereas S2 phosphorylation promotes elongation of Pol II through the gene body and the production of mature mRNA transcript (*8*–*11*). The transcriptional CDKs/cyclins have emerged as compelling targets for transcriptional therapy in cancers (*12*–*14*). However, it is yet unclarified how transcriptional dysregulation finely tuned gene expression processes might stimulate tumor progression and how therapeutical responses can be achieved from global or selective transcriptional perturbation.

A growing body of evidence indicates that biomolecular condensates, formed through liquid-liquid phase separation (LLPS), play an emerging role in organizing the Pol II transcription machinery to enable selective gene regulation (*15*–*19*). Such condensates contribute to processes such as heterochromatin formation, the clustering of enhancer elements, and the concentration of transcription cofactors and long-range enhancer-promoter interactions (*20*–*25*), either promoting or preventing the multistep process of gene activation. Although the importance of specific, structured interactions in transcription is well documented, the role of dynamic, multivalent interactions in condensate-mediated regulation remains poorly understood.

The transcriptional coactivator YAP (Yes-associated protein) is crucial for tissue homeostasis, and its hyperactivation is a hallmark of many cancers (*26*). YAP engage TEA domain transcription factors (TEADs) and drives cancer-specific transcriptional programs that dictate tumor progression and therapeutic resistance (*27*–*31*). Recent studies reveal that YAP hyperactivation results from the formation of nuclear condensates, driven by multivalent interactions, which promote cancer progression. These YAP condensates, which incorporate TEADs and other regulators, facilitate the transcription of proliferation-specific genes (*32*–*36*), highlighting the promising therapeutic opportunities for cancers and other diseases that rely on YAP transcriptional programs (*37*). Given the essential roles of YAP in cancers and limited druggability of YAP as transcriptional cofactor, screening YAP proximal regulators represents a therapeutic strategy for developing cancer treatments (*38*).

Cyclin K (encoded by CCNK) is a new member of the transcriptional cyclin family that form functional complex with two closely related kinases CDK12 and CDK13 (*39*–*41*). They function partially redundantly to regulate Pol II processivity, suppress intronic polyadenylation, ensure proper transcription elongation, and play vital roles in the DNA damage response (DDR) and genomic stability maintenance (*40*, *42*–*44*). Deregulation of CDK12/13 has been implicated in various cancers, and their inhibition has shown therapeutic efficacy. For instance, CDK12 loss sensitizes ovarian cancer cells to PARP inhibitors (*45*, *46*). Although CDK12/13 have been actively studied, the role of Cyclin K beyond being a binding partner remains largely unexplored. Nevertheless, Cyclin K itself has gained increasing attention as a promising therapeutic target (*47*). The development of molecular glue degraders that target Cyclin K to inhibit CDK12/13 demonstrates translational potential for cancer treatment (*48*).

In this study, we uncovered that CDK12/Cyclin K assembles with YAP into nuclear biomolecular condensates via LLPS, forming a hub for YAP phosphorylation at T398. This event sterically hinders inhibitory phosphorylation by LATS kinases on YAP, thereby stabilizing YAP and promoting its oncogenic condensates with TEAD4 to activate target genes. Our study provides the rationale for targeting Cyclin K for YAP transcription–addicted cancers, such as HCC.

## RESULTS

### Elevated Cyclin K expression is linked to HCC progression

**Fig. 1.**
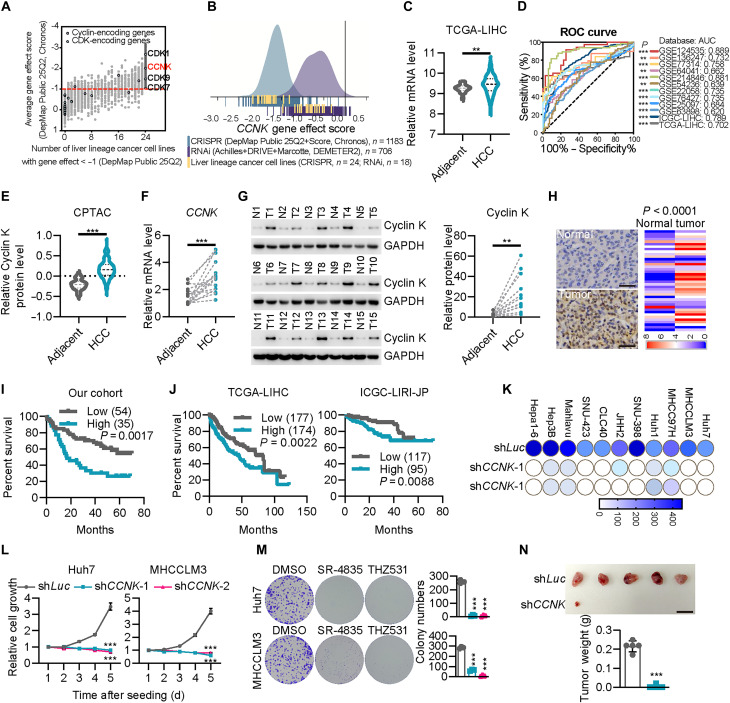
CCNK is essential in multiple cancer types. (**A**) Interrogation of essential genes using CRISPR dependency data from DepMap. (**B**) CCNK is essential across multiple cancer types. (**C**) CCNK is up-regulated in liver cancers (TCGA). (**D**) ROC curves evaluating the diagnostic performance of CCNK expression in 12 independent cohorts of HCC. The area under the curve (AUC) value for each cohort is indicated. (**E**) CCNK is up-regulated in liver cancers (CPTAC). (**F** and **G**) Determination of CCNK mRNA (F) and protein levels (G) in HCC and adjacent tissues (*n* = 15). (**H**) Heatmap demonstration of Cyclin K expression in HCC and adjacent tissues by IHC staining (*n* = 40). Representative IHC images are shown (left). Scale bars, 50 μm. The color scale is the IHC score of CCNK for each normal and tumorous tissue. (**I** and **J**) Prognostic performance of Cyclin K in our cohort (I) and TCGA-LICH and ICGC-LIRI-JP (J). (**K**) Cell viability in CCNK-ablated HCC cell lines. The color scale indicates the number of mean colonies from three independent experiments. (**L**) Cell proliferation analysis in CCNK-ablated HCC cells. Data are presented as means ± SD from one representative experiment. (**M**) Cell viability analysis in HCC cells upon SR-4835 (0.1 μM) and THZ531 (0.1 μM) treatment. DMSO, dimethyl sulfoxide. (**N**) Analysis of tumor growth in CCNK-depleted Huh7 cells. Xenograft weight are quantified and shown (*n* = 5). Gross xenografts are shown. Scale bar, 10 mm. Unpaired *t* test [(C), (E), and (N)], paired *t* test [(F) and (G)], or two-way ANOVA [(L) and (M)] was used to determine statistical significance, respectively. Pearson was used to determine correlation in (H). Data are presented as means ± SD. *P < 0.05, **P < 0.01, and ***P < 0.001.

Cyclins and cyclin-dependent kinases (CDKs) are fundamental regulators of cell cycle progression and transcriptional control, and their dysregulation is a recognized feature of tumorigenesis and progression (*1*, *2*). To investigate their relevance in liver cancer, we analyzed CRISPR dependency data from DepMap (Public 25Q2) across 24 liver lineage cancer cell lines. Four genes, CDK1, CDK7, CDK9, and CCNK, showed consistent essentiality, each with gene effect scores lower than −1 (Fig. 1A). The roles of CDK1, CDK7, and CDK9 in HCC have been extensively studied, whereas the function of CCNK remains largely undefined, providing a rationale for focusing on this gene. We next extended the analysis to the full DepMap dataset, which includes 1183 CRISPR and 706 RNA interference (RNAi) screened cell lines, and observed that CCNK is broadly indispensable across multiple cancer types (Fig. 1B). Transcriptomic data from 12 independent cohorts comprising 1624 HCC samples and 1112 nontumor liver samples [10 Gene Expression Omnibus (GEO) datasets, ICGC-LIHC, and The Cancer Genome Atlas (TCGA)–LIHC] consistently demonstrated significant overexpression of CCNK in liver tumor tissues (Fig. 1C and fig. S1A). Receiver operating characteristic (ROC) curve analyses further indicated that CCNK expression effectively discriminates HCC from the nontumor liver (Fig. 1D). At the protein level, analysis of Clinical Proteomic Tumor Analysis Consortium (CPTAC) data confirmed that Cyclin K abundance is significantly elevated in HCC compared with adjacent nontumor tissues (Fig. 1E). To confirm this in an additional cohort of HCC, we have examined CCNK expression in 15 paired clinical samples and confirmed higher CCNK levels at both the mRNA and protein levels in liver tumor tissues compared to adjacent nontumor tissues (Fig. 1, F and G). Consistently, immunohistochemical (IHC) staining showed an increased expression of Cyclin K in liver cancerous tissues (Fig. 1H). Prognostic evaluation indicated that higher CCNK expression was significantly associated with shorter overall survival in our cohort and in both TCGA and ICGC cohorts (Fig. 1, I and J). Given that CDK12 is the essential binding partner of CCNK, we extended our analysis and observed a similar pattern of up-regulation and association with poor prognosis (fig. S1, B to D). To verify its essentiality in HCC cells, we depleted CCNK expression in 10 human and 1 mouse HCC cell lines and found that CCNK depletion broadly rendered HCC cells inviable (Fig. 1, K and L), as assessed by long-term clonogenic assays and CCK8. Pharmacological inhibition of Cyclin K or CDK12 by SR-4835 and THZ531, respectively, also significantly blunted in Huh7 and MHCCLM3 cells (Fig. 1M). A similar observation was seen in CDK12-ablated HCC cells (fig. S1, E and F). In vitro findings were further corroborated in vivo using a xenograft model, where the growth of tumors with CCNK or CDK12 depletion was markedly impaired (Fig. 1N and fig. S1G). Together, these findings identify CCNK as an essential vulnerability across cancers, including liver cancer, and establish its aberrant overexpression associated with poor prognosis in patients with HCC, providing a strong rationale for further investigation of CCNK in HCC progression.

### Cyclin K stimulates YAP signaling in HCC

**Fig. 2.**
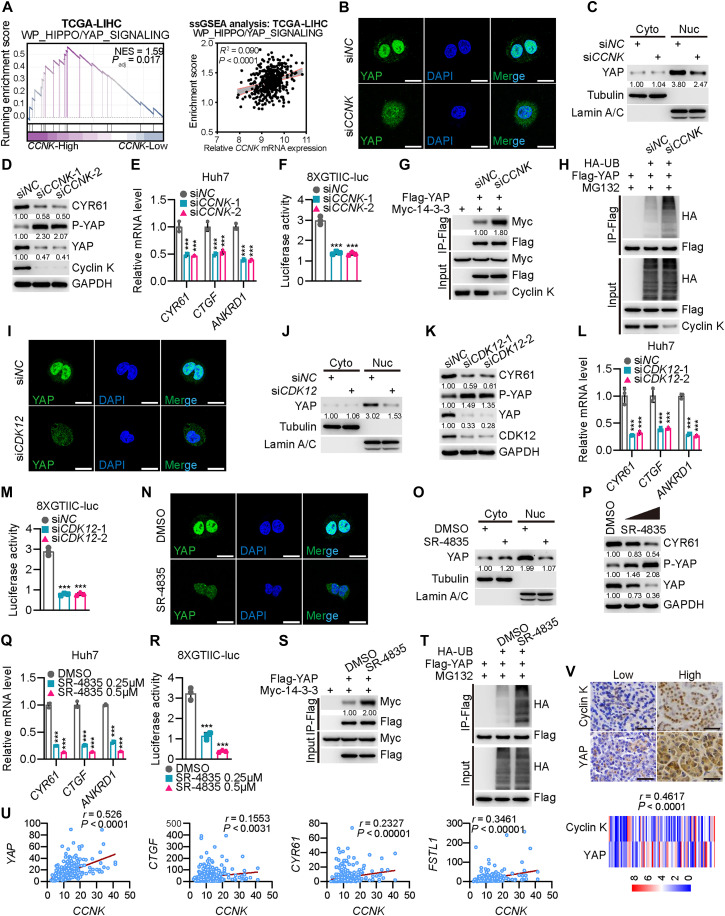
CCNK stimulates YAP activation in HCC. (**A**) YAP signaling is increased in HCC with high CCNK expression. NES, normalized enrichment score. (**B** and **C**) YAP translocates into the cytoplasm in CCNK-depleted cells by immunofluorescence staining (B) and cell fractionation (C). Scale bars, 20 μm. (**D** and **E**) Analysis of YAP signaling in CCNK-depleted Huh7 cells. (**F**) Analysis of TEAD-responsive reporter (8×GTIIC-luc) in HEK-293T cells upon CCNK ablation. (**G** and **H**) Immunoblot and ubiquitination assay evaluating 14-3-3/YAP interaction (G) and ubiquitination (H) in cells with or without CCNK depletion. MG132, 20 μM for 6 hours. (**I** and **J**) Evaluation of YAP subcellular distribution in CDK12-depleted cells. Scale bars, 20 μm. (**K** and **L**) Analysis of YAP activation in cells upon CDK12 loss. (**M**) Analysis of TEAD-responsive reporter in HEK-293T cells with or without CDK12 ablation. (**N** and **O**) Evaluation of YAP localization in Huh7 cells following SR-4835 treatment (0.5 μM) for 8 hours. Scale bars, 20 μm. (**P** and **Q**) Evaluation of YAP activity in cell upon SR-4835 treatment (0.25 and 0.5 μM). (**R**) Analysis of TEAD-responsive reporter in HEK-293T cells treated with or without SR-4835 treatment (0.5 μM) for 8 hours. (**S** and **T**) Evaluation of 14-3-3/YAP interaction and ubiquitination in cells treated with SR-4835 (0.5 μM, 8 hours). MG132, 20 μM for 6 hours. (**U**) Correlation analysis of CCNK expression with YAP and its targets in the TCGA dataset. (**V**) Heatmap demonstration of correlation between Cyclin K and YAP expression in HCC tissues (*n* = 90). Scale bars, 50 μm. The color scale indicates the IHC score of YAP or Cyclin K for each HCC tissue. For [(E), (F), (L), (M), (Q), and (R)], *P* values were determined by one-way ANOVA. For [(U) and (V)], the Pearson’s correlation test was used to analyze the link. Data are presented as means ± SD. *P < 0.05, **P < 0.01, and ***P < 0.001.

To elucidate how CCNK affects HCC cell proliferative activity, signaling pathway enrichment by GSEA (Gene Set Enrichment Analysis) revealed that YAP signaling enrichment was observed in Cyclin K– or CDK12-high expression HCC specimens (Fig. 2A and fig. S2A). CCNK ablation in Huh7 and MHCCLM3 cells induced YAP nuclear exclusion, phosphorylation, and a concomitant reduction in total YAP and its downstream targets, CYR61, CTGF, and ANKRD1 (Fig. 2, B to E, and fig. S2, B and C). Moreover, YAP-responsive luciferase reporter (8xGTIIC-luc) activity was greatly impaired in human embryonic kidney (HEK) 293T cells following CCNK knockdown (Fig. 2F), indicating YAP suppression. Because YAP phosphorylation typically triggered its 14-3-3–mediated ubiquitination and degradation (*26*), we investigated whether CCNK loss inactivates YAP through this canonical pathway. As expected, CCNK loss in cells showed enhanced 14-3-3/YAP interaction and ubiquitination, suggestive of decreased protein stability (Fig. 2G and 2H). To further strengthen these observations, we next asked whether CDK12 depletion phenocopies the effect of CCNK loss. CDK12 knockdown similarly promoted YAP cytoplasmic sequestration, phosphorylation, and suppression of its transcriptional activity (Fig. 2, I to M, and fig. S2, D and E). In contrast to the effects of their depletion, we showed that expression of either CCNK or CDK12 stimulated YAP nuclear retention in Huh7 cells with high cell density (fig. S2F). CCNK overexpression potently facilitated YAP activation in HCC cells and HEK-293T and impaired YAP binding to 14-3-3 and reduced YAP ubiquitination (fig. S2, G to L), which was similarly observed in HCC cells upon CDK12 overexpression (fig. S2, M to P). Using two structurally distinct inhibitors—SR-4835 and THZ531, which target CCNK and CDK12 (*48*), respectively, we demonstrated that nanomolar concentrations effectively recapitulated the phenotypic effects of genetic depletion in both Huh7 and MHCCLM3 cells (Fig. 2, N to T, and fig. S2, Q to U). To provide clinical relevance of the CDK12/CCNK-YAP signaling axis in HCC, our analysis of HCC datasets revealed a significant positive correlation between the mRNA expression of CCNK and that of YAP and its key transcriptional targets, including CYR61, CTGF, and FSTL1 (Fig. 2U and fig. S2V). We further validated this relationship at the protein level through IHC grading of a tissue microarray (TMA), which demonstrated a notable coexpression of YAP with Cyclin K (Fig. 2V). Collectively, these results demonstrate that CDK12/Cyclin K augments YAP signaling in HCC.

### CDK12/Cyclin K binds to YAP via the PPxY motif

**Fig. 3.**
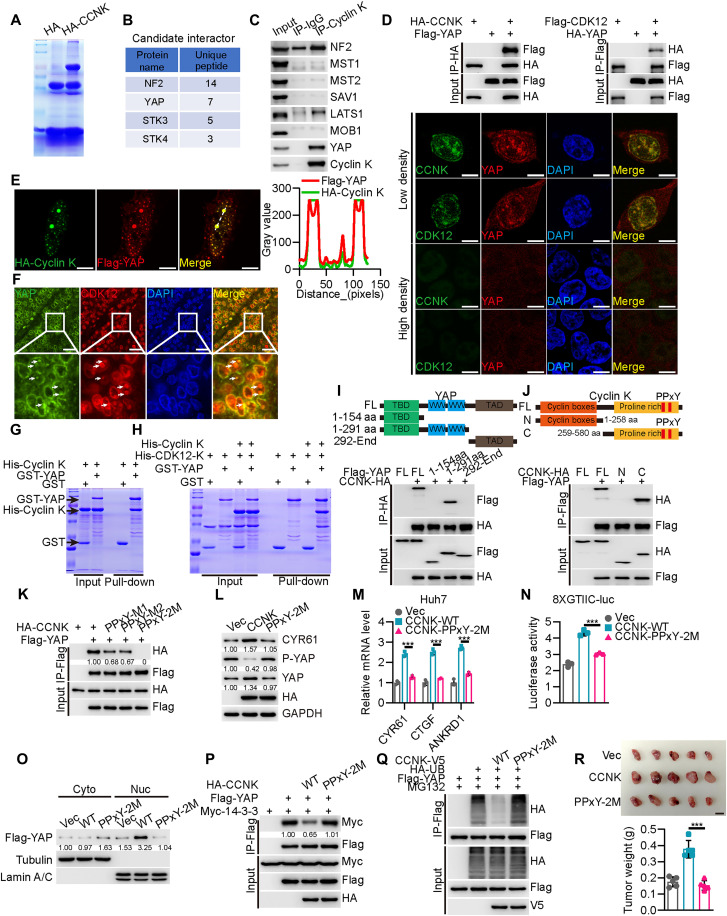
CDK12/Cyclin K physically associates with YAP. (**A**) Coomassie Brilliant Blue staining showing Cyclin K immunoprecipitates. (**B**) List of candidate interactors related to canonical YAP signaling. (**C**) Co-IP of endogenous Cyclin K and Hippo signaling components. (**D**) YAP interacts with Cyclin K and CDK12. (**E**) Immunostaining showing the colocalization of YAP and Cyclin K in Huh7 cells. Left: Huh7 cells transfected with exogenous HA-Cyclin K and Flag-YAP. Scale bars, 10 μm. Quantification of the fluorescence intensity along the dashed line indicated in the merged image is shown (middle). Right: Costaining of endogenous YAP with Cyclin K or CDK12 in Huh7 cells with low or high density. Scale bars, 10 μm. (**F**) Immunostaining for YAP and CDK12 in human HCC tissues. Scale bars, 10 μm. (**G**) GST pull-down assay of in vitro interaction between Cyclin K and YAP. Arrows indicate the proteins at the expected molecular weight. (**H**) Cyclin K was required for YAP/CDK12 interaction. (**I** and **J**) Mapping the region(s) of YAP or Cyclin K that mediates the interaction. Diagram shows the domains of YAP or Cyclin K protein. aa, amino acids. (**K**) Cyclin K interacts with YAP via two PPxY motifs. Individual PPxY mutant, PPxY-M1 or PPxY-M2. Double mutant, PPxY-2M. (**L** to **N**) YAP activity or TEAD-responsive reporter analysis in Huh7 cells with vector, WT CCNK, or PPxY-2M. (**O**) Cell fractionation evaluating YAP localization in Huh7 cells with vector, WT CCNK, and PPxY-2M. (**P** and **Q**) Determination of 14-3-3/YAP interaction and YAP ubiquitination in HEK-293T cells with vector, WT CCNK, or PPxY-2M. (**R**) Quantification of xenograft weight in indicated groups (*n* = 5). Gross images of xenografts are shown. Scale bar, 5 mm. For [(L), (M), and (Q)], *P* values were determined by one-way ANOVA. Representative data are presented as means ± SD. *P < 0.05, **P < 0.01, and ***P < 0.001.

To clarify how Cyclin K regulates YAP signaling at molecular level, we profiled its binding partners through coimmunoprecipitation (Co-IP) and liquid chromatography–mass spectrometry (LC-MS) in Huh7 cells infected with vector control or hemagglutinin (HA)–CCNK (Fig. 3A). Although initial screening identified several bona fide Hippo pathway components (YAP, MST1/2, and NF2), only YAP was validated as a CCNK binding partner (Fig. 3, B and C). We used exogenous Co-IP assays and further substantiated the formation of a complex between YAP, Cyclin K, and CDK12 (Fig. 3D and fig. S3A), albeit with differential binding intensity. In agreement with this, both exogenous and endogenous YAP exhibited strong nuclear colocalization with and Cyclin K or CDK12 in Huh7 cells (Fig. 3E). Clinically, we observed that CDK12 and YAP were colocalized in HCC tissues (Fig. 3F). This observation was further supported by a glutathione *S*-transferase (GST) pull-down assay, indicating a direct interaction between the YAP and Cyclin K (Fig. 3G). Given that YAP interacts with both CDK12 and Cyclin K, we speculate that YAP interacts with CDK12 via Cyclin K. We demonstrated that Cyclin K was essential for the CDK12/YAP interaction in vitro (Fig. 3H), and this interaction was completely abolished in cells following depletion of endogenous Cyclin K (fig. S3B). To map the specific molecular domains required for this interaction, we generated a series of truncation mutants encoding various domains of both YAP and Cyclin K. Co-IP analysis revealed that both the WW domains of YAP were essential for its association with Cyclin K whereas the C-terminal region of Cyclin K (amino acids 270 to 580) mediates its binding to YAP (Fig. 3, I and J). Notably, the C terminus of Cyclin K harbors two conserved PPxY (where x is any amino acid) motifs, well known as a WW domain chaperone that mediates many protein-protein interactions. This is particularly prevalent within the Hippo pathway. Encouraged by this finding, we next sought to determine whether these PPxY motifs mediate the interaction between Cyclin K and YAP. Our experiments revealed that mutation of either PPxY motif (designated PPxY-M1 and PPxY-M2) modestly reduced the Cyclin K/YAP interaction, whereas mutation of both motifs (PPxY-2 M) completely abolished it (Fig. 3K). We next investigated whether the PPxY motifs of Cyclin K are required for the YAP activation and oncogenic activity driven by Cyclin K. As anticipated, mutation of the PPxY motifs greatly blunted Cyclin K–mediated YAP dephosphorylation, stabilization, nuclear retention, and transcriptional activity compared with wild-type (WT) Cyclin K (Fig. 3, L to Q, and fig. S3, C to E). indicating that CCNK/CDK12 stimulate YAP signaling via its association with YAP by the PPxY domain. Given that YAP and its paralog TAZ are the major transcriptional coactivators of Hippo signaling and both harbor WW domains, we thus determine whether TAZ is affected by CDK12/CCNK as well. CCNK bound to TAZ via the PPxY domain and stimulates TAZ nuclear retention and protein levels in HCC cells (fig. S3, F to I). In summary, this confirms that both YAP and TAZ are coordinately regulated by CCNK/CDK12. Consistent with these in vitro findings, subcutaneous xenografts derived from Huh7 cells expressing the PPxY-2M mutant grew significantly slower than those expressing WT Cyclin K, at a rate comparable to control cells (Fig. 3R). Moreover, CDK12-mediated YAP activation requires an intact CDK12/Cyclin K complex as the CDK12-L996F mutation significantly impaired the activation of YAP signaling (fig. S3, J to L). These results demonstrate that the PPxY motifs of Cyclin K are essential for the Cyclin K/YAP association and for YAP activation both in vitro and in vivo.

### CDK12/Cyclin K undergoes phase separation with YAP

**Fig. 4.**
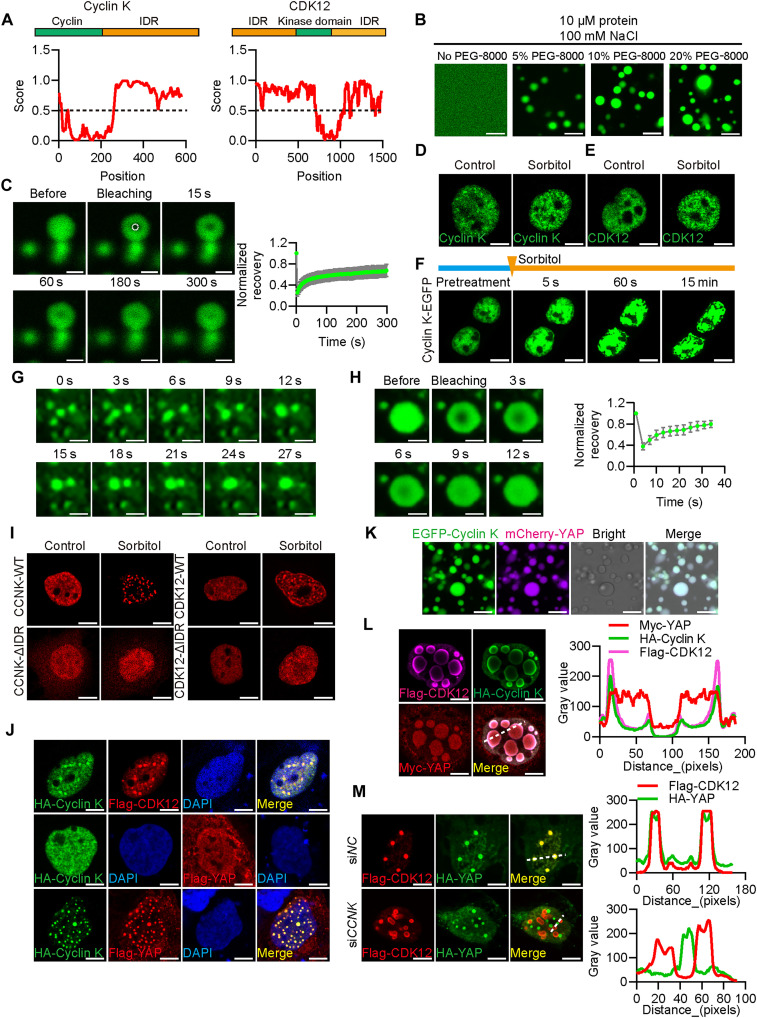
YAP co-occupies CDK12/Cyclin K LLPS. (**A**) IDR prediction across Cyclin K and CDK12 proteins. (**B**) Purified EGFP-CCNK before (left) and after (middle) the addition of indicated concentration of PEG-8000. The liquid droplets were visualized using fluorescence microscopy. Scale bars, 5 μm. (**C**) FRAP images of EGFP-CCNK condensates. Representative images were acquired after photobleaching the indicated ROI with fluorescence signal recovery curves. The dotted circle indicates the region of photobleaching. Scale bars, 2 μm. (**D** and **E**) Endogenous Cyclin K and CDK12 formed condensates in Huh7 cells were treated with 0.2 M sorbitol for 20 min. Scale bars, 10 μm. (**F**) CCNK-EGFP formed nuclear foci after sorbitol treatment for indicated times. Scale bars, 20 μm. (**G**) Time-lapse imaging of the fusion of nuclear CCNK-EGFP droplets. Scale bars, 1 μm. (**H**) FRAP images of nuclear CCNK-EGFP condensates. Scale bars, 1 μm. Quantification of the FRAP curve of CCNK-EGFP condensates are shown (*n* = 7). (**I**) Confocal images of Huh7 cells expressing either WT CCNK (left) and CDK12 (right) or an IDR region truncated protein with or without sorbitol treatment (0.2 M) for 20 min. Scale bars, 10 μm. (**J**) Representative images of CDK12 and Cyclin K condensates (top) and individual CCNK, YAP (middle), or both CCNK/YAP (bottom). Nuclei were counterstained with DAPI. Scale bars, 10 μm. (**K**) Purified EGFP-CCNK and mCherry-YAP proteins were incubated with 20% PEG-8000. Images were acquired under fluorescence and bright-field illumination. Scale bars, 5 μm. (**L**) Condensates of Flag-CDK12, HA-CCNK, and Myc-YAP in Huh7. Quantification of fluorescence intensity along the dashed line indicated in the merged image is shown. Scale bars, 5 μm. (**M**) Condensates of CDK12 and YAP in Huh7 cells with or without Cyclin K depletion. Scale bars, 10 μm. Data are presented as means ± SD.

As clearly seen in Fig. 3 (E and F), Cyclin K likely formed liquid-like condensate with YAP, and we therefore test this concept. Given that intrinsically disordered regions (IDRs) are essential for LLPS, we analyzed the IDRs of CDK12 and Cyclin K using online prediction tools, including IUPred3, PONDR, and PLAAC. All tools predicted with high confidence that both proteins harbor extensive IDRs (Fig. 4A). We then asked whether Cyclin K has an intrinsic ability to form condensates independently. To this end, we purified recombinant enhanced green fluorescent protein (EGFP)–Cyclin K–His and mCherry-YAP-His proteins for in vitro LLPS assays. Confocal microscopy revealed that EGFP–Cyclin K formed liquid droplets in vitro in the presence of various concentrations of PEG-8000 (polyethylene glycol, molecular weight 8000) (Fig. 4B). Time-lapse imaging showed that EGFP–Cyclin K droplets readily recovered within minutes, further confirming its ability to form dynamic liquid-like condensates (Fig. 4C). To extend these findings to a cellular context, we observed that both endogenous Cyclin K formed small condensates in the nucleus of Huh7 cells upon sorbitol treatment (Fig. 4D). Similar to Cyclin K, its partner CDK12 also formed nuclear puncta without sorbitol treatment (Fig. 4E). Moreover, the puncta were formed within 1 min after sorbitol treatment in Huh7 cells with EGFP-CCNK expression (Fig. 4F). Consistent with the in vitro finding, EGFP–Cyclin K droplets fused over time and displayed rapid fluorescence recovery after photobleaching (FRAP) in Huh7 cells upon sorbitol treatment (Fig. 4, G and H), collectively indicating that Cyclin K has the intrinsic ability to form condensates. The IDRs were essential for CDK12/Cyclin K phase separation as either Cyclin K or CDK12 that lacked this domain was unable to undergo phase separation regardless of sorbitol treatment (Fig. 4I). Having previously established YAP’s capacity for phase separation, we next test whether Cyclin K incorporates YAP into the condensates. Compared to the individual expression of Cyclin K or YAP, their coexpression led to mutual recruitment into the phase-separated condensates. Similar reciprocal recruitment was observed between CDK12/Cyclin K (Fig. 4J). Consistently, Cyclin K and YAP were recruited reciprocally into the liquid-like condensates in vitro (Fig. 4K). Notably, under normal osmotic conditions, CDK12, Cyclin K, and YAP strongly colocalized within condensates (Fig. 4L). Given that Cyclin K interacts with both CDK12 and YAP, we hypothesized that it mediates the formation of CDK12/YAP condensates. Upon depletion of Cyclin K in Huh7 cells, YAP was almost excluded from CDK12 condensates, despite the observation that the formation of their individual condensates remained unaffected (Fig. 4M). Collectively, these results indicate that these components assemble into a tightly associated functional signaling complex.

### CDK12/Cyclin K complex phosphorylates YAP at threonine-398

**Fig. 5.**
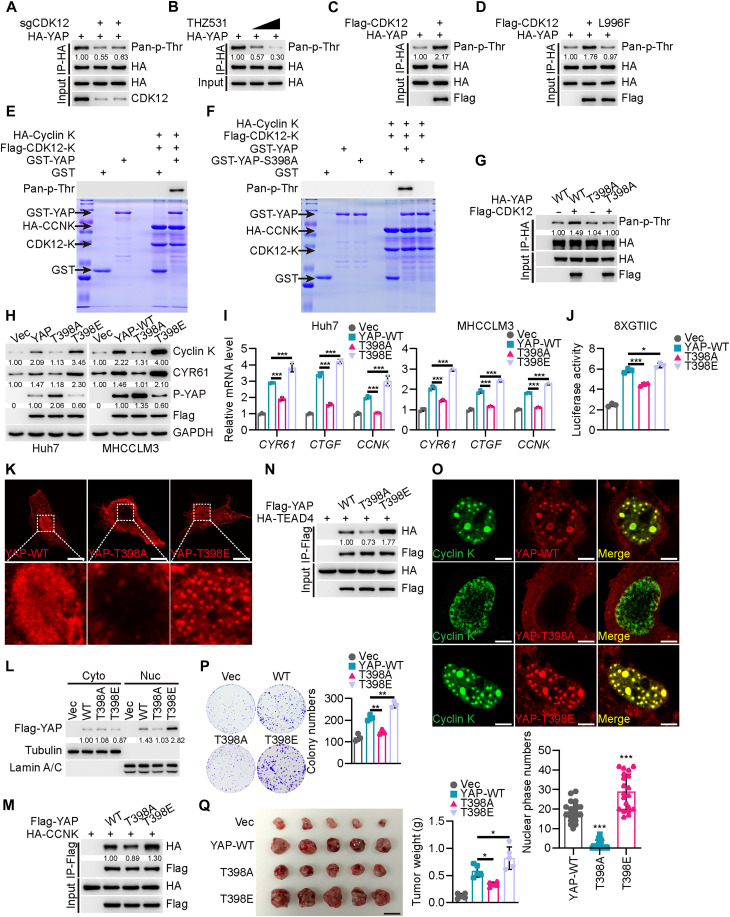
CDK12/Cyclin K phosphorylates YAP. (**A** and **B**) Determination of total YAP phosphorylation by pan-Phospho-(Thr) (pan-p-Thr) antibody. Exogenous YAP from HEK-293T cells infected with sgCDK12 lentiviral particles (A) or treated with THZ531 (0.1 and 0.25 μM) (B) was precipitated to detect its phosphorylation levels. (**C**) Determination of YAP phosphorylation in HEK-293T cells with or without CDK12 overexpression. (**D**) Determination of total YAP phosphorylation in HEK-293T cells following vector, WT CDK12, or CDK12-L966F overexpression. (**E**) Purified GST-YAP was incubated with active CDK12/Cyclin K in the presence of SAM in vitro. YAP phosphorylation was detected by immunoblot. (**F**) Purified GST-YAP or GST-S398A was incubated with active CDK12/Cyclin K in the presence of SAM in vitro. (**G**) Examination of YAP phosphorylation in 293T cells transfected with CDK12 along with YAP or YAP-T398A. (**H** to **J**) Evaluation of YAP signaling activation in HCC cells by immunoblot (H), qPCR (I), and reporter activity (J) analysis. (**K**) Immunostaining analysis of YAP and indicated mutants localization in Huh7 cells transfected with YAP, YAP-T398A, and YAP-T398E. Scale bars, 20 μm. (**L**) Analysis of YAP distribution by cell fractionation in Huh7 cells. (**M** and **N**) YAP-T398 phosphorylation stimulated its interaction with Cyclin K (M) and TEAD4 (N). (**O**) CCNK condensates with YAP or indicated mutants. Scale bars, 7.5 μm. Quantification of merged puncta per cell is shown. (**P**) Assessment of cell proliferative capacity in Huh7 cells infected with vector, YAP, YAP-T398A, and YAP-T398E. Representative images and the quantification are shown. (**Q**) Gross image and quantification of xenografts derived from Huh7 cells infected with vector, YAP, YAP-T398A, and YAP-T398E (*n* = 5). Scale bar, 10 mm. For [(I), (J), (O), (P), and (Q)], *P* values were determined by one-way ANOVA. Representative data are presented as means ± SD. *P < 0.05, **P < 0.01, and ***P < 0.001.

Given the kinase activity of CDK12 and modulation of YAP activation, we ask whether CDK12 regulates its activation through direct phosphorylation. Because the CDK family kinases phosphorylate proline-based serine or threonine, we sought to determine whether CDK12 manipulation affects the overall YAP phosphorylation status using Phospho-(Ser/Thr) antibody. CDK12 knockout or pharmacological inhibition of CDK12 in cells impaired the total phosphorylation level of YAP (Fig. 5, A and B), whereas overexpression of CDK12, but not the CDK12^L996F^ that perturbs Cyclin K binding, increased its YAP phosphorylation (Fig. 5, C and D).

In an in vitro kinase assay, YAP was phosphorylated by purified CDK12/Cyclin K (Fig. 5E). Using liquid chromatography–tandem mass spectrometry (LC-MS/MS), we further identified the phosphorylation of threonine-398 (T398). Mutating T398 to Ala abolished such phosphorylation (Fig. 5, F and G), suggesting that CDK12 phosphorylates T398 of YAP. To further determine whether CDK12 regulates YAP activity via this phosphorylation, we found that YAP-T398E was more potent, whereas YAP-T398A was ineffective, in stimulating YAP transcriptional activity (Fig. 5, H to J). In addition, YAP-T398A in Huh7 cells showed more cytoplasmic distribution whereas YAP-T398E was largely localized in the nucleus (Fig. 5, K and L), as assessed by immunofluorescence staining and cell fractionation. Consistently, phosphodefective YAP-T398A mutation disrupted its association with Cyclin K, unlike the WT YAP and phosphomimetic YAP-T398E mutant (Fig. 5M). As YAP primarily signals through TEAD transcription factors, we ask whether YAP phosphorylation stimulates its transcriptional activity via enhanced YAP/TEAD binding. YAP-T398A perturbed whereas YAP-T398E favored its association with TEAD4 (Fig. 5N).

Consistently, the YAP and YAP-T398E mutant were efficiently incorporated into Cyclin K condensates (Fig. 5O), indicating that phosphorylation-regulated dynamic phase separation of YAP associated with Cyclin K that modulates YAP activity.

In line with this, YAP^T398E^ stimulated HCC cell proliferation, whereas YAP-T398A YAP^T398A^ lost this ability (Fig. 5P). Last, we confirmed the function of CDK12-mediated YAP phosphorylation in vivo that xenografts from Huh7 cells expressing YAP-T398E were bigger than WT xenografts, whereas YAP-T398A delayed xenograft growth (Fig. 5Q). Together, these results demonstrate that CDK12/Cyclin K promote YAP oncogenic activation by phosphorylating YAP at T398.

To address whether condensate formation is required for CCNK-mediated YAP phosphorylation, we generated an additional CCNK mutant with IDR deletion but an intact PPxY domain (termed as ΔIDR+PPxY). This did not form condensates itself upon sorbitol treatment (fig. S3M) yet preserved the ability to associate with YAP, compared with the PPxY-2M mutant (fig. S3N). The PPxY-2M mutant and YAP formed condensates separately, whereas ΔIDR+PPxY mutant was incorporated into YAP condensates upon sorbitol treatment (fig. S3O). However, either PPxY-2M or ΔIDR+PPxY mutant lost its ability to stimulate YAP T398 phosphorylation and activate YAP signaling and target gene expression (fig. S3, P to S).

### CDK12/Cyclin K is transcriptionally activated by YAP signaling

To investigate the mechanism driving Cyclin K up-regulation in HCC, we analyzed its promoter region to identify potential transcription factor binding motifs. We identified two or three canonical YAP/TEAD binding motifs (5′-CATTCC-3′) within the CCNK or CDK12 promoter, suggesting that CCNK/CDK12 may be transcriptionally regulated by YAP. As expected, YAP overexpression significantly stimulated the expression of CCNK as well as its canonical targets, including CTGF and CYR61, in Huh7 and MHCCLM3 cells (fig. S4, A and B). Notably, YAP also promoted Ser^2^ and Ser^5^ phosphorylation of Pol II, which are catalyzed by the CDK12/Cyclin K complex. Conversely, small interfering RNA (siRNA)–mediated depletion of YAP/TAZ or TEAD1/3/4 greatly reduced CCNK expression (fig. S4, C and D). Moreover, treatment of HCC cells with metabolic cues that modulate YAP signaling, such as serum-borne lysophosphatidic acid (LPA) and glucose deprivation, yielded consistent results (fig. S4, E and F), indicating that CCNK is induced upon YAP signaling alteration. To determine whether YAP/TEAD directly stimulates CCNK transcription, we performed chromatin immunoprecipitation followed by quantitative polymerase chain reaction (ChIP-qPCR). These assays confirmed that TEAD4 binds specifically to the first motif (M1) within the CCNK promoter. A known TEAD4 target region in the CTGF promoter served as a positive control and was significantly enriched (fig. S4, G and H).

We then generated a CCNK promoter-driven luciferase reporter to assess its transcriptional activity. Knockdown of YAP alone, or simultaneous knockdown of YAP and TEAD4 in HEK-293T cells, markedly reduced reporter activity, which was abolished when using a reporter plasmid containing mutated YAP/TEAD binding sites (fig. S4I). On the contrary, YAP or coexpression of YAP and TEAD4 remarkably stimulated the WT CCNK but not the mutant reporter activity (fig. S4J). To further strengthen this conclusion, analysis of public ChIP sequencing datasets derived from liver tissues confirmed changes in CCNK promoter enrichment by TEAD4 (fig. S4K). Together, these data here demonstrate that CCNK is a bona fide target of YAP/TEAD4 signaling. In addition, elevated CDK12 and CCNK levels in TP53-mutant HCC suggest a potential regulatory role of TP53 in modulating CDK12/CCNK (fig. S4L).

### CDK12/Cyclin K complex competes with LATS kinases for YAP phosphorylation

**Fig. 6.**
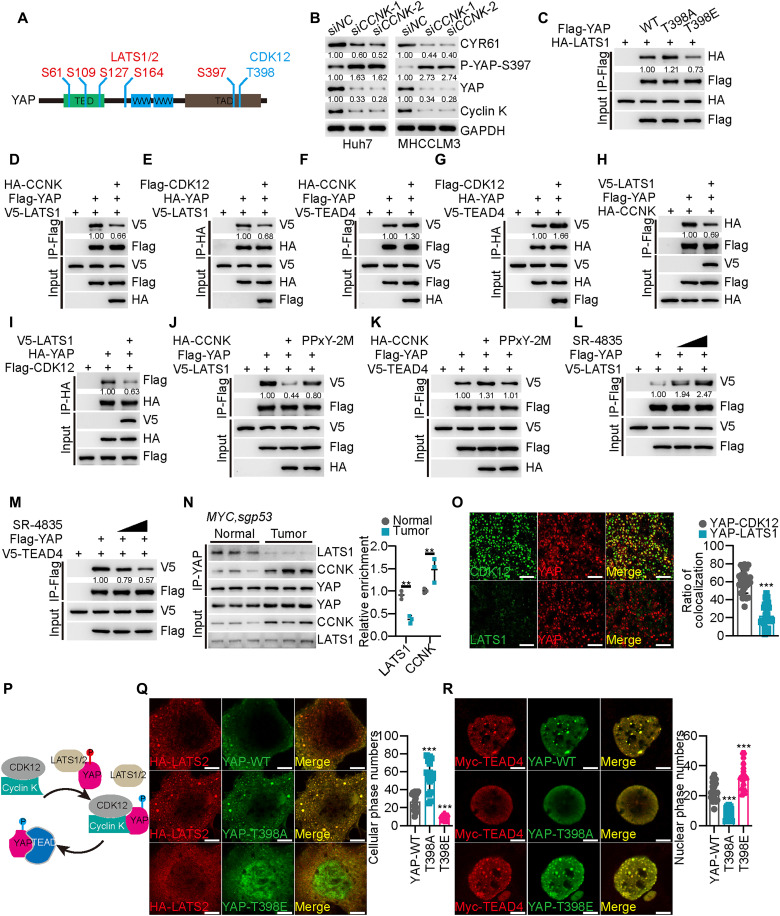
CDK12/Cyclin K compete with LATS1/2 kinases for YAP phosphorylation. (**A**) Diagram for YAP domains and phosphorylation sites. The canonical LATS kinases phosphorylation sites and CDK12 phosphorylation site were indicated in red and blue, respectively. (**B**) Assessment of YAP S397 phosphorylation in Huh7 and MHCCLM3 cells upon CCNK depletion. (**C**) Effects of YAP T398 phosphorylation on its interaction with LATS1. HEK-293T cells transfected with vector, YAP, YAP-T398A, and YAP-T398E along with LATS1 were subjected to Co-IP and immunoblotting. (**D** to **G**) CDK12/Cyclin K overexpression perturbed LATS1/YAP interaction (D and E) and enhanced YAP/TEAD4 interaction (F and G). (**H** and **I**) Evaluation of YAP association with Cyclin K (H) or CDK12 (I) in the presence of LATS1. (**J** and **K**) Determination of YAP binding to LATS1 (J) or TEAD4 (K) in the presence of control, CCNK, and CCNK-PPxY-2M. (**L** and **M**) Evaluation of YAP/LAST1 (L) and YAP/TEAD4 binding (M) in cells treated with SR-4835 (0.25 and 0.5 μM for 12 hours). (**N**) Analysis of YAP interaction with CDK12 or LATS1 in mouse HCC and adjacent tissues (*n* = 3). (**O**) Colocalization analysis of YAP with CDK12 or LATS1 in human HCC tissues. Ten fields of view from three tissue sections were randomly selected, and colocalization of YAP with CDK12 and LATS1 was analyzed using the Saiviewer-2.1.1 software. Representative images are shown. Scale bars, 60 μm. (**P**) Schematic representation hypothesizing the potential acting mechanism of CDK12/Cyclin K in stimulating YAP via competition with toward LATS kinases. (**Q** and **R**) Immunofluorescence showing the condensates of LATS2 (Q) or TEAD4 (R) with YAP and indicated mutants in Huh7 cells. Quantification of merged puncta per cell is shown. Scale bars, 7.5 μm. For (Q) and (R), *P* values were determined by one-way ANOVA. Data are presented as means ± SD. *P < 0.05, **P < 0.01, and ***P < 0.001.

It is worth noting that YAP is predominantly suppressed via five major phosphorylation sites by the canonical LATS kinases (Fig. 6A). We therefore asked whether CDK12-mediated phosphorylation at T398 competes with LATS kinase–dependent phosphorylation. To investigate this, we first assessed the phosphorylation levels S397, one of the five residues adjacent to T398 with commercial antibody. As expected, Cyclin K knockdown in Huh7 and MHCCLM3 cells markedly increased YAP S397 phosphorylation (Fig. 6B). Consistent with this observation, YAP-T398A displayed a stronger interaction with LATS kinases compared to WT YAP and YAP-T398E (Fig. 6C and fig. S5A). These results suggest a potential competitive relationship between CDK12-mediated phosphorylation at T398 and LATS-mediated phosphorylation. The presence of either Cyclin K or CDK12 impaired the association between YAP and LATS kinases (Fig. 6, D and E, and fig. S5, B and C), thereby strengthening the YAP-TEAD4 interaction (Fig. 6, F and G). The observation that verteporfin, a known compound that disrupts YAP/TEAD4 association, had no effect on YAP/CCNK interaction indicates that CCNK acts through YAP to modulate YAP signaling output (fig. S5, D and E). Conversely, LATS kinases markedly reduced the amount of CDK12 or Cyclin K coimmunoprecipitated with YAP (Fig. 6, H and I, and fig. S5, F and G). However, mutation of both PPxY motifs (PPxY-2M) in YAP, which disrupts its binding to Cyclin K, largely abolished this inhibitory effect on YAP-LATS binding (Fig. 6, J and K, and fig. S5H).

Furthermore, treatment with the Cyclin K inhibitor SR-4835 dose-dependently enhanced YAP-LATS binding while reducing YAP-TEAD4 interaction (Fig. 6, L and M, and fig. S5I). Consistent with this, SR-4835 was able to reverse the dissociation of YAP from LATS kinases induced by either Cyclin K or CDK12 (fig. S5, J to M). Similarly, the enhanced YAP-TEAD4 association resulting from CDK12/Cyclin K expression was attenuated upon SR-4835 treatment (fig. S5, N and O). To support the proposed mechanism, we examined the prevalence of the CDK12/CCNK/YAP and LATS/YAP complexes in tumors and adjacent tissues from a MYC/Trp53-induced HCC model. Notably, YAP was more frequently associated with CDK12/CCNK in tumor tissues, whereas the opposite pattern was observed in adjacent normal tissues (Fig. 6N). Furthermore, immunostaining of human HCC samples revealed greater colocalization of YAP with CDK12 than with LATS1 (Fig. 6O). These findings suggest a predominant CDK12/CCNK/YAP signaling in HCC (Fig. 6P).

To support this notion further, we determined the condensation of YAP to LATS kinases and TEADs. Whereas YAP and YAP-T398A were largely cytoplasmic and formed condensates with LATS2, only YAP-T398E was excluded from LATS2 condensates and exhibited nuclear retention, suggestive of active YAP signaling (Fig. 6Q). Consistently, coexpression of TEAD4 with YAP led to robust nuclear co-condensation, which was particularly enhanced with the YAP-T398E mutant. In contrast, Huh7 cells coexpressing TEAD4 and YAP-T398A showed minimal condensate formation (Fig. 6R). To strengthen this further, we test whether TEADs are functionally required for CDK12/CCNK-mediated effects downstream YAP. TEAD1/3/4 depletion significantly reduced YAP levels and generally abrogated YAP activation, target gene expression, and reporter activity induced by CCNK in HCC and HEK-293T cells (fig. S5, P to R). In a xenograft model, TEAD1/3/4 depletion canceled CCNK-stimulated tumor growth (fig. S5S). Collectively, these data establish a regulatory switch between CDK12/Cyclin K and LATS kinases for YAP binding and phosphorylation, which dictates YAP activity and its transcriptional output.

To test the potency of T398 phosphorylation for YAP activity, we have evaluated the activity of YAP, YAP-T398E, and YAP-S127A in vitro and in vivo. YAP-T398E was more potent than WT YAP but was less effective than YAP-S127A (fig. S6, A to C). Similarly, tumors derived from Huh7 cells expressing YAP-T398E were larger than those expressing WT YAP, confirming its protumorigenic effect. However, these tumors remained smaller than those formed by cells expressing the S127A mutant (fig. S6D). This supports a model in which phosphorylation at T398 serves as a regulatory modulation that operates within the canonical Hippo signaling.

### Targeting Cyclin K for treating CDK12/Cyclin K–YAP signaling axis–addicted HCC

**Fig. 7.**
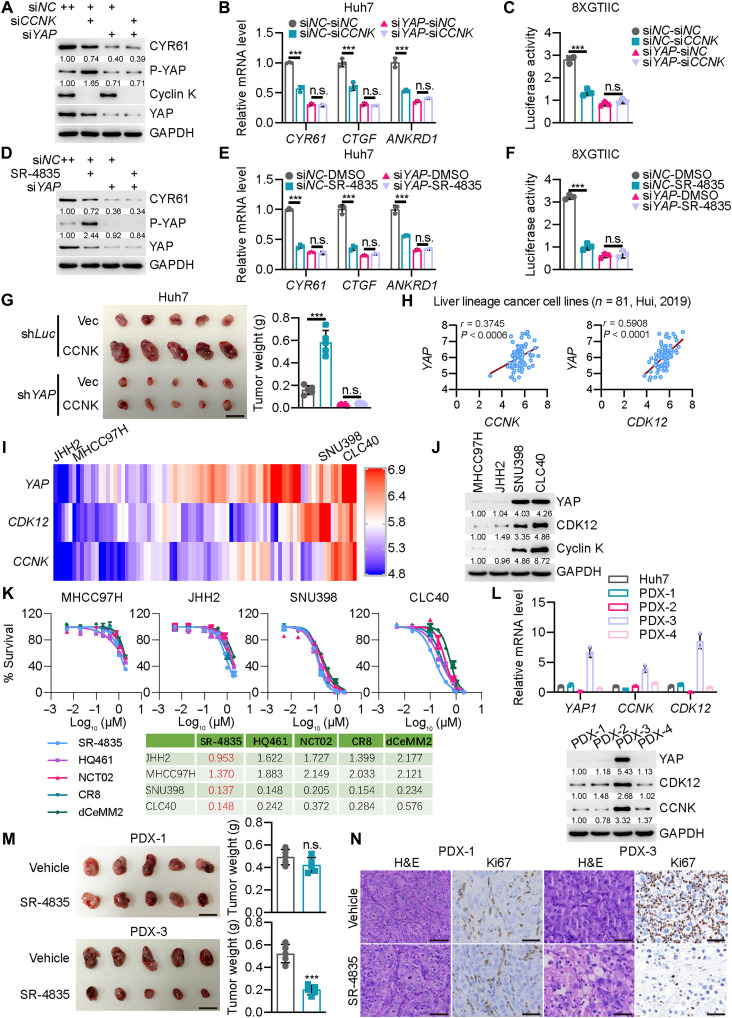
Targeting CDK12/Cyclin K–YAP signaling addiction provides therapeutic vulnerability in HCC. (**A** to **C**) Control or YAP-depleted Huh7 cells were transfected with or without siRNA targeting CCNK and subjected to immunoblotting (A), qPCR analysis (B), and reporter activity analysis (C). (**D** to **F**) Control or YAP-depleted Huh7 cells were treated with or without SR-4835 and subjected to immunoblotting (D), qPCR analysis (E), and reporter activity analysis (F). (**G**) Control or YAP-depleted Huh7 cells with or without CCNK overexpression were implanted into nude mice. Gross image and quantification of xenograft weight are shown. Scale bar, 10 mm. (**H**) Correlation analysis of YAP with CCNK or CDK12 in HCC cell lines. Transcriptional datasets were download from the GEO (GSE97098). The Pearson’s correlation test was used to analyze the link. (**I**) Cluster analysis of CDK12, CCNK, and YAP coexpression. JHH2, MHCC97H, SNU398, and CLC40 cell lines were indicated. The color scale indicates the level of each gene presented as reads per kilobase per million mapped reads (RPKM). (**J**) Protein levels of CCNK, YAP, and CDK12 in indicated HCC cell lines. (**K**) Quantification of cellular sensitivity (IC_50_) to Cyclin K molecular glue degraders in indicated cell lines. IC_50_ values are shown. (**L**) Determination of the mRNA and protein levels of CCNK, YAP, and CDK12 in Huh7 and PDX models. (**M**) Evaluation of tumor growth of vehicle and SR-4835 (5 mg/kg per day) treatment in the PDX mouse model. Gross xenografts and quantification of xenograft weight are shown. Scale bars, 10 mm. (**N**) Histological analysis of xenografts from indicated PDX models. Scale bars, 50 μm. For [(B), (C), (E), (F), and (G)], *P* values were determined by one-way ANOVA. The unpaired *t* test was used in (M) to determine statistical significance. Data are presented as means ± SD. *P < 0.05, **P < 0.01, and ***P < 0.001; n.s., not significant.

Having established that the CDK12/Cyclin K complex critically controls YAP signaling and transcriptional output in HCC, we next investigated whether this function is dependent on YAP itself. Biochemical and in vivo assays consistently demonstrated that YAP was an essential downstream effector of oncogenic CDK12/Cyclin K signaling and was required for HCC xenograft progression in nude mice (Fig. 7, A to G, and fig. S7, A to G). This mechanistic and functional link between CDK12/Cyclin K and YAP signaling suggests that inhibiting this complex could be a viable therapeutic strategy for treating the CDK12/Cyclin K–YAP–addicted HCC subtype. To explore this further, we analyzed the correlation between CDK12/Cyclin K and YAP expression across 81 authenticated liver cancer cell lines. As anticipated, both Cyclin K and CDK12 expression showed a strong positive correlation with YAP levels (Fig. 7H). We therefore hypothesize that a therapeutic window may exist for targeting CDK12/Cyclin K in HCC exhibiting simultaneous hyperactivation of YAP signaling and high expression of CDK12/Cyclin K. We then stratified HCC cell lines according to their coexpression of CDK12/Cyclin K and YAP, selecting two models with low YAP activity (JHH2 and MHCC97H) and two models exhibiting YAP addiction (CLC40 and SNU398) for further analysis (Fig. 7I). The protein expression of CDK12, Cyclin K, and YAP in these selected cell lines was verified (Fig. 7J) before evaluating their sensitivity to a series of Cyclin K inhibitors. Despite diverse structures, CLC40 and SNU398 cells showed higher sensitivity to all Cyclin K glue degraders within the nanomolar range, whereas low to modest response was observed in JHH2 and MHCC97H cells (Fig. 7K), suggesting a concept that high coexpression of CDK12/Cyclin K and YAP dictates their drug sensitivity to CDK12/Cyclin K inhibitor. To proof this further, we used patient-derived xenografts (PDXs) of HCC to evaluate the antitumoral efficacy of Cyclin K inhibitors. The expression of YAP, Cyclin K, and CDK12 were determined in four different PDX mouse models. In this small cohort of PDX samples, we observed a trend where high expression of CDK12, Cyclin K, and YAP proteins co-occurred in one of the four samples (Fig. 7L). Given the relatively lower IC_50_ (median inhibitory concentration) of SR-4835 among all Cyclin K glue degraders tested, we have selected SR-4835 for in vivo study. To minimize potential off-target effects, we used a lower dose of SR-4835 than previously reported (*49*). Following established tumor growth, mice from PDX-1 and PDX-3 models were treated with SR-4835 or a vehicle control. Notably, tumors derived from the PDX-1 model showed limited inhibition, whereas those from the PDX-3 model exhibited significant repression (Fig. 7, M and N). To provide functional relevance of CDK12/CCNK-mediated YAP phosphorylation in this process, we compared the therapeutic response to Cyclin K inhibitors of in MHCC97H and SNU398 with WT YAP, YAP-T398A, and T398E. To this end, we found that YAP-T398E, but not YAP-T398A, resulted in resistance to Cyclin K glue degraders in MHCC97H cells where YAP signaling was activated and could not be inhibited further by CCNK MGDs. However, YAP-WT showed increased sensitivity CCNK MGDs, possibly due to CCNK up-regulation via the YAP/CCNK feedback mechanism, which could be suppressed by CCNK MGDs. A similar, although less pronounced, change was observed in SNU398 cells upon expression of T398E compared with MHCC97H (fig. S7, H to I). Consistently, treatment with verteporfin, a known inhibitor of YAP-TEAD interaction, rendered SNU398 cells resistant to SR-4835, and this effect was less significant in MHCC97H cells (fig. S6J), indicating that the therapeutic response to Cyclin K inhibitors in HCC cells is dependent on the CCNK/YAP-TEAD signaling axis. Collectively, these results demonstrated that a functional CDK12/Cyclin K–YAP in YAP-addicted HCC could be effectively treated through targeting CDK12/Cyclin K.

## DISCUSSION

In cancer, transcriptional addiction, the pathological dependence of tumor cells on specific transcriptional programs, is a fundamental driver of malignancy (*1*, *3*, *5*). In HCC, this state is often orchestrated by the transcriptional coactivator YAP, which, upon hyperactivation, engages TEAD transcription factors to propel the expression of pro-proliferative and prosurvival genes (*50*–*52*). However, how the general transcriptional program integrates with specific factors like YAP to drive oncogenic phenotypes in cancer remains to be investigated.

In the work, we have revealed that the profound dependency of a wide array of HCC cell lines on CCNK, validated by both genetic ablation and pharmacological inhibition, underscored its nonredundant role in maintaining HCC cell viability, positioning it as both a prognostic biomarker and a therapeutic vulnerability. The observation that Cyclin K, but not its kinase partners CDK12 or CDK13, is broadly essential across multiple cancer types suggests that CDK12 and CDK13 likely have redundant functions in maintaining cell viability, including HCC. Although CDK12 and CDK13 share most of their substrates implicated in critical regulators of RNA processing and Pol II, numerous studies have described their selective and nonoverlapping roles for CDK12 and CDK13 and differential phosphorylation events as a result of their individual inhibition (*42*–*44*, *46*). Although we have not addressed the potential involvement of CDK13 in YAP activation, it will be an interesting work in the future.

Our data link the essentiality of Cyclin K to its role in the multifaceted activation of YAP signaling, thereby identifying a critical oncogenic axis and therapeutic dependency on the CDK12/Cyclin K–YAP pathway in HCC. Mechanistically, the CDK12/Cyclin K complex undergoes LLPS, forming a biomolecular condensate that acts as a regulatory hub. Within this hub, CDK12 directly phosphorylates YAP at the novel T398 site. The presence of Cyclin K is indispensable for this process as it bridges the interaction between CDK12 and YAP; in its absence, CDK12 cannot associate with YAP in vitro, and YAP is excluded from CDK12 condensates in cells. This integrated mechanism, coupling enzymatic activity with biomolecular condensation, finely tunes YAP activity and its transcriptional output.

By concentrating CDK12 and YAP within these nuclear puncta, Cyclin K likely enhances the kinetics of YAP phosphorylation while simultaneously sequestering it from inhibitory kinases like LATS1/2, the major kinases that strictly repress YAP (*26*, *27*). The observation that the phosphomimetic YAP^T398E^ mutant is more efficiently incorporated into Cyclin K condensates suggests that phosphorylation by CDK12 may reinforce phase separation, implying a feedforward loop that amplifies YAP signaling.

This notion is supported by the competitive relationship between CDK12-mediated T398 phosphorylation and LATS-mediated phosphorylation, a potential “tug-of-war” mechanism that dictates YAP’s fate. We here showed that, by phosphorylating the adjacent T398 residue, CDK12/Cyclin K appears to sterically hinder LATS kinase access to YAP, thereby stabilizing YAP and promoting its oncogenic function. Consistent with this finding, the YAP-T398A mutant efficiently localizes to LATS2 condensates, in stark contrast to the YAP-T398E mutant, which is largely excluded. This illustrates a general concept of how the CDK12/Cyclin K–regulated Hippo signaling hub is organized within cells via biomolecular condensation. Notably, YAP functions as a signaling hub integrated by multiple kinases, including AMPK, c-Abl, CK1δ/ε, and SRC, in addition to the canonical LATS kinases (*53*). These regulators typically phosphorylate YAP in the cytoplasm or during nucleocytoplasmic shuttling, leading to its cytoplasmic sequestration and inactivation. Our study reveals that the CDK12/CCNK complex operates differently by phosphorylating YAP within the nucleus to maintain its nuclear retention and transcriptional activity. Thus, our work identifies CDK12/CCNK as an additional nuclear layer of control that fine-tunes YAP’s transcriptional output, complementing the canonical cytoplasmic “on-off” switch governed by upstream kinases.

Expectedly, the proposed mechanism drives YAP transcriptional output. Specifically, TEAD4 readily forms condensates with YAP and does so even more efficiently with the YAP-T398E mutant in the nucleus, whereas the YAP-T398A mutant does not. These collectively indicate that the CDK12/Cyclin K complex directs multivalent YAP/TEAD4 phase-separated condensates to potentiate YAP activity. It should be noted that coexpressing exogenous TEAD4 and YAP leads to efficient YAP nuclear retention and colocalization, supporting the newly identified role of TEADs in driving YAP/TAZ transcriptional condensates in the nucleus (*36*), although this has not been extensively investigated. TEAD4 also promotes nuclear retention of the YAP-T398A mutant, despite its predominant cytoplasmic localization when expressed alone and the subsequent formation of only limited TEAD4/YAP-T398A condensates. The mechanism by which TEAD4 bypasses the CDK12/Cyclin K signal toward YAP remains an important question for future investigation.

We have identified a positive feedback loop in which YAP/TEAD transcriptionally up-regulates CCNK. This self-reinforcing circuit amplifies YAP signaling to dynamically respond to diverse oncogenic clues, which is commonly observed in multiple pathways, including the Hippo signaling (*54*). This mechanism may partially explain the aberrant CCNK expression observed in HCC. Furthermore, CDK12, the kinase partner of Cyclin K (CCNK), also appears to be a YAP/TEAD target gene, suggesting extensive cross-talk between YAP and CDK12/Cyclin K signaling. Notably, both CCNK and CDK12 are markedly up-regulated in TP53-mutant HCC compared to TP53-WT tumors, implying that TP53 might also contribute to CDK12/Cyclin K expression and potential activation, warranting further investigation in the future.

The stratification of HCC cell lines and PDXs based on coexpression of CDK12/Cyclin K and YAP, and the correlation with sensitivity to Cyclin K inhibitors, provides an effective biomarker strategy for patient selection. The potent antitumor effects of SR-4835 in YAP high signature PDX models offer a strong preclinical foundation for exploring this targeted therapy in clinical trials. Future work should also focus on developing more specific and potent inhibitors and validating these biomarkers in prospective clinical studies.

In conclusion, this study redefines the CDK12/Cyclin K complex as a critical oncogenic circuit that co-opts phase separation and competitive phosphorylation to hyperactivate YAP signaling for therapeutic vulnerability, establishing CDK12/Cyclin K as a promising therapeutic target for a molecularly defined subset of HCC.

## MATERIALS AND METHODS

### Sample collection

Liver cancer TMAs containing 90 pathologically examined cases were collected from Renji Hospital between 2014 and 2018 with the understanding and written consent of each participant. Of these, 40 paired liver cancer and adjacent tissues were assembled separately. All procedures were performed under the approval of the Ethics Committee of the Research Ethics Committee of Renji Hospital, School of Medicine, Shanghai Jiao Tong University (approval no. RA-2020-352). Surgical liver cancer samples used for PDX generation were collected from therapy-naive patients who underwent surgery in Renji Hospital School of Medicine, Shanghai Jiao Tong University. All research was conducted in accordance with both the Declarations of Helsinki and Istanbul.

### PDX and xenograft model

PDXs were generated as previously described by transplanting fresh fragments subcutaneously into nude recipient mice (Passage 1, P1). Five days postimplanting, recipient mice bearing PDXs were randomly assigned to treatment with vehicle or SR-4835 (orally 5 mg/kg once a day) for 21 days according to approved protocols [approval no. 2025-ky-011(K)]. Mice were housed in a pathogen-free environment at Tongji University and treated according to approved protocols (no. 2025-0162). For tumor growth test in vivo, a total of 5 × 10^6^ Huh7 cells suspended in 100 μl of serum-free Dulbecco’s modified Eagle’s medium (DMEM) was subcutaneously injected into the upper flank of each mouse. Three weeks after tumor inoculation, mice were euthanized for analysis of tumor weight. All animals received humane care according to the criteria outlined in the “Guide for the Care and Use of Laboratory Animals.”

### Cell culture, DNA construct transduction, and RNAi

HEK-293T and Huh7 cell lines were originally obtained from the American Type Culture Collection (ATCC) and cultured in DMEM high glucose medium (BasalMedia Technologies) supplemented with 10% (v/v) fetal bovine serum (FBS; FSD500, ExCellbio Inc.) at 37°C in a humidified incubator containing 5% CO_2_. Hep3B, MHCCLM3, MHCC97H, Huh1, SNU-398, SNU-423, Mahlavu, and Hepa1-6 were obtained from the Cell Bank of Shanghai Institutes of Biological Sciences and maintained in DMEM high glucose medium. CLC40 cells was originally prepared by H. Lijian and was purchased from BIO-RESEARCH INNOVATION CENTRE SUZHOU, Shanghai Institute of Biochemistry and Cell Biology, Center for Excellence in Molecular Cell Science, Chinese Academy of Sciences. CLC40 cells were maintained in RPMI 1640 medium supplemented with epidermal growth factor (EGF) (40 ng/ml), Insulin-Transferrin-Selenium (ITS) (10%, v/v) and Y-27632 (10 μM). All cell lines underwent detection for mycoplasma contaminants using a Mycoplasma Detection Kit (40611ES25, YEASEN, Shanghai, China) and were confirmed to be mycoplasma negative. For expression of DNA constructs, lentiviral plasmid or short hairpin RNA (shRNA) lentiviral particles were prepared as previously described (*55*). Cells were infected with lentiviral particles as indicated for subsequent experimental procedures. siRNAs targeting YAP/TAZ, CDK12, CCNK, and TEAD1/3/4 and a negative control were synthesized by Shanghai GenePharma Co. Ltd. (Shanghai, China). siRNA oligos were transfected into HCC cells using Lipofectamine RNAiMAX Transfection Reagent (13778150, Thermo Fisher Scientific) according to the manual provided. For overexpression of CDK12, HCC cells were infected with adenovirus particles carrying CDK12. The siRNA, lentiviral shRNA, or single guide RNA (sgRNA) target sequences for human cells are provided below:

siYAP: 5′-GGUGAUACUAUCAACCAAATT-3′

siTAZ: 5′-GCGAUGAAUCAGCCUCUGAAT-3′

siTEADs: 5′-GATCAACTTCATCCACAAGCT-3′

siCCNK-1: 5′-CCACCAAATCCTGGATCTTTA-3′

siCCNK-2: 5′-GCAGACCATCAAGTTTGATTT-3′

siCDK12-1: 5′-GGCCTGATGTTATCAAACTTT-3′

siCDK12-2: 5′-GCCCAATTCAGAGAGACATTT-3′

shCDK12-1: 5′-CCGGGCTCGGCTCTATAACTCTGAACTCGAGTTCAGAGTTATAGAGCCGAGCTTTTTG-3′

shCDK12-2: 5′-CCGGGCACTGAAAGAGGAGATTGTTCTCGAGAACAATCTCCTCTTTCAGTGCTTTTTG-3′

shCCNK-1: 5′-CCGG CCACCAAATCCTGGATCTTTACTCGAGTAAAGATCCAGGATTTGGTGG TTTTTG-3′

shCCNK-2: 5′-CCGG GCAGACCATCAAGTTTGATTTCTCGAGAAATCAAACTTGATGGTCTGC TTTTTG-3′

shTEAD1/3/4: 5′-CCGGGATCAACTTCATCCACAAGCTCTCGAGAGCTTGTGGATGAAGTTGATCTTTTTG-3′

sgCDK12-1: 5′-TGGCCTTCAAACTAGACCGA-3′

sgCDK12-2: 5′-ACTGACCGACTGCCTTCTCG-3′

### CCK8 and colony formation assay

A total of 2 × 10^3^ cells infected with indicated lentivirus or transfected with indicated constructs were plated into 96-well plates in 200 μl. Cell viability was measured by CCK8 daily for 5 days. For colony formation, a total of 500 cells were seeded into 6-well plates and cultured for 15 days. Colonies were fixed by 4% paraformaldehyde, stained using 0.005% crystal violet, counted, and analyzed.

### Reagents and antibodies

DMEM high glucose was purchased from Shanghai BasalMedia Technologies (China). NCT02 (HY-W181530), SR-4835 (HY-130250), HQ461 (HY-144981), THZ531 (HY-103618), CR8 (HY-18340), and dCeMM2 (HY-144971) were obtained from MedChemExpress (Shanghai, China). GST Agarose Resin (20507ES10) and Ni-NTA Agarose Resin (20502ES10) were purchased from Yeasen Biotechnology (Shanghai, China). Antibodies used for immunoblot against CDK12 (26816-1-AP, 1:1000), CYR61 (1:1000, 26689-1-AP), GAPDH (60004-1-Ig, 1:5000) and MYC tag (60003-2-I, 1:5000), V5 tag (14440-1-AP, 1:5000), LATS1 (17049-1-AP, 1:1000), LATS2 (20276-1-AP, 1:1000), SAV1 (32277-1-AP, 1:1000), MST1 (22245-1-AP, 1:10,000), MST2 (66637-1-Ig, 1:10,000), and NF2 (21686-1-AP, 1:1000) were purchased from Proteintech (Wuhan, China). Cyclin K (A10261, 1:1000), MOB1 (A23963, 1:5000), Pan Phospho-Serine/Threonine (AP1067, 1:1000), α-Tubulin (A6830, 1:5000), anti HA-Tag (AE008, 1:5000), β-Actin (AC026, 1:5000), HRP-conjugated Goat anti-Rabbit IgG (H+L) (AS014, 1:5000), and HRP-conjugated Goat anti-Mouse IgG (H+L) were obtained from ABclonal Inc. FLAG M2 gel (A2220), mouse anti-FLAG antibody and anti-GST tag (G7781, 1:5000), TAZ (T4077, 1:1000), and YAP (1:1000, WH0010413M1) antibodies were purchased from Sigma-Aldrich. Phospho-YAP(S127) (4911, 1:1000) and Phospho-YAP(S397) (13619, 1:1000) was purchased from Cell Signaling Technology Inc. Anti-CCNK (HPA000645, 1:100) for immunostaining was purchased from Sigma-Aldrich. Cyclin K (A10261, 1:100) for IHC staining was obtained from ABclonal Inc.

### Luciferase reporter assay

A 0.5-μg 8xGTIIC-luciferase reporter plasmid (Addgene_34615) was individually cotransfected with a Renilla vector, with or without the indicated control or plasmids into HEK-293T cells. After 24 hours, cells were harvested for detection. Dual-Luciferase Assay kit (Promega) was used to measure the luciferase activity on a GloMax 20/20 luminometer (Promega) following the manufacturer’s instructions. For preparation of the CCNK promoter luciferase reporter, the DNA sequence of the CCNK promoter containing TEAD4 binding sites were amplified by PCR and subcloned to the pGL3-promoter vector.

### Immunofluorescence and histological staining

For immunofluorescence staining, Huh7 cells were fixed in 4% paraformaldehyde for 30 min, washed in phosphate-buffered saline (PBS), permeabilized with 0.1% Triton X-100 for 30 min, and blocked with 3% bovine serum albumin (BSA) in PBS for 30 min. This was followed by incubation with primary antibodies Flag (1:500, Novus, NBP1-06712SS), Mouse anti Flag mAb (1:1000, AE005), Rabbit anti Flag mAb (1:1000, AE169PM), Mouse HA (1:1000, Sigma-Aldrich, H3663), HA-Tag Rabbit mAb (1:1000, AE105), YAP (1:500, Sigma-Aldrich, WH0010413M1), CDK12 (26816-1-AP, 1:500), and Anti-CCNK (HPA000645, 1:200). The coverslips or sections were then incubated with appropriate fluorescence-conjugated secondary antibodies: Anti-Mouse IgG-Atto 594 (1:500, 76085-1ML-F, Sigma-Aldrich), Anti-Rabbit IgG-Atto 594 (1:500, 77671-1ML-F, Sigma-Aldrich), Anti-Mouse IgG-Atto 488 (1:500, 62197-1ML-F, Sigma-Aldrich) or Anti-Rabbit IgG-Atto 488 (1:500, 18772-1ML-F, Sigma-Aldrich), and Goat Anti-Rat IgG H&L Alexa Fluor 647 (1:500, ab150167, Abcam) for 1 hour at room temperature in the dark. Nuclei were counterstained with DAPI (4′,6-diamidino-2-phenylindole; G1407, ServiceBio). Images were acquired with an upright fluorescence microscope (Olympus, BX63, Japan). Tissues were fixed in 4% formalin and embedded in paraffin for hematoxylin and eosin (H&E) staining. For IHC staining, tumor sections or TMA sections were deparaffinized, rehydrated, followed by heat-induced antigen retrieval, and incubated overnight at 4°C with the indicated antibodies against YAP (1:100, Proteintech, 13584-1-AP), Cyclin K (A10261, 1:200), CDK12 (26816-1-AP, 1:200), and Ki67 (1:10,000, Proteintech, 28074-1-AP). IHC staining was independently assessed by two experienced pathologists and scored as previously reported.

### Plasmid construction and protein expression and purification

For plasmid construction, CDK12-3xFlag was cloned into pAV-CMV-P2A-GFP (WZ Biosciences Inc.). CDK12 kinase domain (termed as CDK12-k-3xFlag, amino acids 714 to 1063) was cloned into pLenti-puro. 3xFlag-YAP, Myc-14-3-3, V5-TEAD4, V5-LATS1, 3xHA-LATS1, 3xHA-LATS2, CCNK-3XHA, CCNK-V5, CCNK-PPxY-2M-3XHA [PPxY→AAAA at positions PPAY (amino acids 462 to 465) and PPTY (amino acids 510 to 513)], and ΔIDR+PPxY-3XHA (amino acids 1 to 257 and 462 to 580) were cloned into pCDH-MCS-T2A-Puro-MSCV (System Biosciences, CD522A-1). CCNK-V5, CCNK-ΔIDR-3XHA (amino acids 1 to 257), CCNK-C-3XHA (amino acids 462 to 580), CDK12-ΔIDR-Flag (amino acids 722 to 1037), and YAP truncations were cloned into pcDNA3.1. pcDNA3.1-HA-UB was purchased from Miaoling Biology (China). pcDNA3.1-CCNK-3xHA was a gift from T. Han from the National Institute of Biological Sciences, China. CDK12-L996F-3xFlag, YAP-T398A-3xFlag, and YAP-T398E-3xFlag were generated by site-directed mutagenesis using PCR. GST-YAP was cloned into pGEX-4 T-1. EGFP-CCNK-6xHis, mCherry-YAP-6xHis, and EGFP-TEV CCNK-6xHis were cloned into pET28-MBP-TEV-EGFP-His with minor modification.

The GST-YAP, EGFP-CCNK-6xHis, mCherry-YAP-6xHis, and EGFP-TEV CCNK-6xHis fusion proteins were expressed in *Escherichia coli* BL21 (DE3) cells and purified as previously described (*56*). Briefly, protein expression was induced with 100 mM isopropyl-β-d-thiogalactopyranoside (IPTG) in LB medium at 18°C under vigorous shaking until the OD_600_ (optical density at 600 nm) reached ~0.8. Cells were harvested, resuspended in ice-cold PBS or lysis buffer [50 mM tris-HCl (pH 7.5), 500 mM NaCl, 20 mM imidazole, and 5% glycerol], and lysed by high-pressure homogenization. The GST-YAP protein was affinity purified using Glutathione Sepharose 4B resin and eluted with elution buffer [50 mM tris-HCl, 15 mM glutathione, and 20 mM dithiothreitol (DTT) (pH 8.0)], whereas the His-tagged proteins were purified using Ni-NTA resin and eluted with lysis buffer supplemented with 200 mM imidazole. All protein preparations were subsequently subjected to gel filtration chromatography on a Superdex 200 10/300 GL column (GE Healthcare, Chicago, USA). Last, the purified proteins were concentrated using 30 -kDa molecular weight cut-off (MWCO) Ultra Centrifugal filter (Amicon). The maltose-binding protein (MBP) tag was cleaved to generate the EGFP-CCNK-6xHis, mCherry-YAP-6xHis, and CCNK-6xHis proteins using Tobacco Etch Virus (TEV) protease. Specifically, the reaction was performed in TEV Protease Reaction Buffer [50 mM tris-HCl (pH 7.5), 0.5 mM EDTA, and 1 mM DTT] with TEV Protease added at a ratio of 1 unit per 2 μg of MBP-fusion protein.

### In vitro kinase assay

Active CDK12/CCNK complex were purified similarly as previously reported (*57*). Briefly, Flag-CDK12 and CCNK-HA plasmids were cotransfected into 10-cm dishes of HEK-293T cells for 24 hours. HEK-293T cells expressing Flag-CDK12/CCNK-HA were lysed in radioimmunoprecipitation assay (RIPA) buffer, incubated with 50 μl of Flag M2 gel and 50 μl of anti-HA affinity gel overnight on a gentle rotator. After extensive wash, Flag-CDK12/CCNK-HA protein complexes were eluted with 500 μl of the HA peptide (5 mg/ml) and Flag peptide (5 mg/ml) in TBS [10 mM tris-HCl and, 150 mM NaCl (pH 7.4)]. The eluates were concentrated using 30-kDa molecular weight cutoff ultrafiltration spin columns. Flag-CDK12/CCNK-HA proteins were quantified by the BCA protein quantification kit (E112-01, Vazyme, China). To identify potential phosphorylation sites of YAP by CDK12/CCNK, 2 μg of recombinant GST-YAP purified from *E. coli* was incubated with 2 μg of Flag-CDK12/CCNK-3xHA purified from HEK-293T cells in kinase assay buffer [25 mM tris-HCl, 5 mM beta-glycerophosphate, 2 mM DTT, 0.1 mM Na_3_VO_4_, 10 mM MgCl_2_, and 0.15 mM adenosine triphosphate] at 30°C for 30 min. The reaction mixtures were incubated for 30 min at 30°C, terminated with SDS sample buffer, subjected to SDS–polyacrylamide gel electrophoresis (PAGE), and analyzed by LC-MS/MS.

### Immunoprecipitation and pull-down assay

For the purification of the Cyclin K protein complex, Huh7 cells infected with either a control or a CCNK-HA lentiviral particles was lysed in RIPA buffer. The lysates were incubated with 40 μl of anti-HA affinity gel overnight on a gentle rotator. The gel was then washed five times with RIPA buffer, and the bound complexes were eluted with 500 μl of the HA peptide (5 mg/ml) in TBS [10 mM tris-HCl and 150 mM NaCl (pH 7.4)]. The eluates were concentrated using 3-kDa molecular weight cutoff ultrafiltration spin columns and analyzed by SDS-PAGE, Coomassie blue staining, and LC-MS/MS. Pull-down assays were performed by incubating 10 μg of recombinant GST or GST-YAP with 10 μg of His-CCNK or His-CDK12 (amino acids 727 to 1020, CSB-EP882147HUc7, CUSABIO, China) in RIPA buffer overnight with 20 μl of GST Agarose Resin. Beads were washed five times, and bound proteins were eluted by boiling in SDS-loading buffer, separated by SDS-PAGE, and stained with Coomassie blue or analyzed by immunoblotting.

### LC-MS/MS analysis, database search, and protein identification

Protein samples from immunoprecipitation or in vitro kinase assay were separated by SDS-PAGE, followed by Coomassie blue staining, in-gel trypsin digestion, peptide extraction, and LC-MS/MS analysis, as previously described (*58*). Briefly, The peptides were resuspended with 4 μl of solvent A (0.1% formic acid), and subsequently 1 μl of the loading sample was separated by a reversed-phase chromatography column (Inopticks, PepMap C18, 1.6 μm, 250 mm by 75 μm). Last, the online analysis was performed with quadrupole time-of-flight mass spectrometer (timsTOF Pro, Bruker, GRE). The column flow rate was 0.2 μl/min. The linear gradient for standard protein was 3 to 26% phase B (acetonitrile with 0.1% formic acid) in 0 to 50 min, 26 to 42% B (50 to 55 min), 45 to 85% B (55 to 57 min), and 85% B for 3 min.

Survey full-scan mass spectra were acquired across the mass range of 100 to 1700 *m/z* (mass/charge ratio) in positive electrospray mode, and the accumulation and ramp time was 100 ms. The single-cycle acquisition period was 1.16 s, including one full thermal ionization mass spectrometry (TIMS) scan and 10 parallel accumulation serial fragmentation (PASEF) MS/MS scans. During the PASEF MS/MS scans, the collision energy increased linearly as a function of mobility, from 20 eV at 1/*k*_0_ = 0.6 V·s/cm^2^ to 59 eV at 1/*k*_0_ = 1.6 V·s/cm^2^. Other MS parameters were set as follows: The intensity threshold was 5000; the range of 1/*k*_0_ was 0.75 to 1.37 V·s/cm^2^; the capillary voltage was 1500 V; the auxiliary gas flow rate was 3 liters/min; the ionization temperature was 200°C; the column temperature was 55°C. All the mass spectral data generated by Nano-HPLC-MS/MS were online searched using PEAKS ONLINE (X Build, version 1.4.2020-10-02_113407) against the UniprotKB/SwissProt database (Taxonomy: *Homo sapiens*, 20,420 entries). The precursor mass error tolerance was set to 20 ppm (parts per million), the fragment mass error tolerance was set to 0.05 Da, the maximum allowed missed cleavages of specific trypsin digestion was set to 2, and the length of the identified peptides was set to 6 to 45 amino acids. The variable modifications were set to oxidation (on M +15.995 Da), phosphorylation (on STY +79.966 Da), and acetylation (Protein N-term +42.011 Da). The false discovery rate (FDR) for peptide and protein group was set to less than 0.01.

### In vitro phase separation experiments

EGFP-CCNK-his or mCherry-YAP-his was expressed in BL21, purified by Ni-NTA Agarose Resin, and resolved in 20 mM Tris and 100 mM NaCl (pH 8) buffer. Purified EGFP-CCNK or mCherry-YAP recombinant proteins (10 μM) without or with different doses (% w/v) of PEG-8000 for indicated times was gently transferred onto a 35-mm glass-bottom dish for live imaging using a confocal laser scanning microscope (LSM 880, Zeiss) fitted with a Plan-Apochromat ×10/0.45 M27 objective (2.0-mm working distance).

### Live imaging and FRAP analysis

Huh7 cells infected with lentiviral EGFP-CCNK particles were seeded at a confluency of −20% on a 35-mm glass-bottom dish. Cells were treated without or with 0.2 M sorbitol for indicated times for live imaging and FRAP experiments. Live cells were visualized under a confocal microscope (A1Rsi, Nikon) using a ×100 oil-immersion objective. The EGFP-CCNK condensates were photobleached using a 488-nm laser beam. Bleaching was focused on a circular region of interest (ROI) with 100% laser power, and time-lapse images were acquired at 3-s intervals over 4 min after photobleaching. Images were processed with the ImageJ software.

### Survival and pathway enrichment

The log-rank test was applied to compare the overall survival or disease-free times between indicated groups stratified by indicated genes, and Kaplan-Meier survival curves were plotted using Prism 10. Gene set variation analysis (GSVA) was used to obtain enrichment scores of CCNK and CDK12.

### Statistical analysis

Data were presented as means ± SD, and statistical analysis was performed in Prism 10 (GraphPad Software Inc.). Comparisons between two groups were performed using an unpaired two-tailed Student’s *t* test. A one-way analysis of variance (ANOVA) followed by Dunnett’s or Bonferroni’s multiple comparisons test was used to compare more than two groups. The correlation was determined by the Pearson correlation test.
