# Supplementary material for: Cyclin K condensates bridge CDK12 to phosphorylate and drive oncogenic YAP activation in hepatocellular carcinoma
Source: Sci Adv. 2026 Jul 3;12(27):eaec6492. doi: 10.1126/sciadv.aec6492 (PMC13330896; doi:10.1126/sciadv.aec6492)
Supplement: Supplementary file 1 — Figs. S1 to S7 Legends for tables S1 to S3 [file sciadv.aec6492_sm.pdf]

Science Advances
AAAS

Supplementary Materials for

Cyclin K condensates bridge CDK12 to phosphorylate and drive oncogenic YAP activation in hepatocellular carcinoma

Yang Sun *et al.*

Corresponding author: Yang Sun, yangsun0927@163.com; Ming Zhan, mzhan@coh.org; Qiwei Li, liqiwei@renji.com; Hongcheng Wang, whc72988@126.com; Yonglong Zhang, yonglz@sjtu.edu.cn, yonglongzhang@sibcb.ac.cn

Sci. Adv. **12**, eaec6492 (2026)
DOI: 10.1126/sciadv.aec6492

The PDF file includes:

Figs. S1 to S7
Legends for tables S1 to S3

Other Supplementary Material for this manuscript includes the following:

Tables S1 to S3

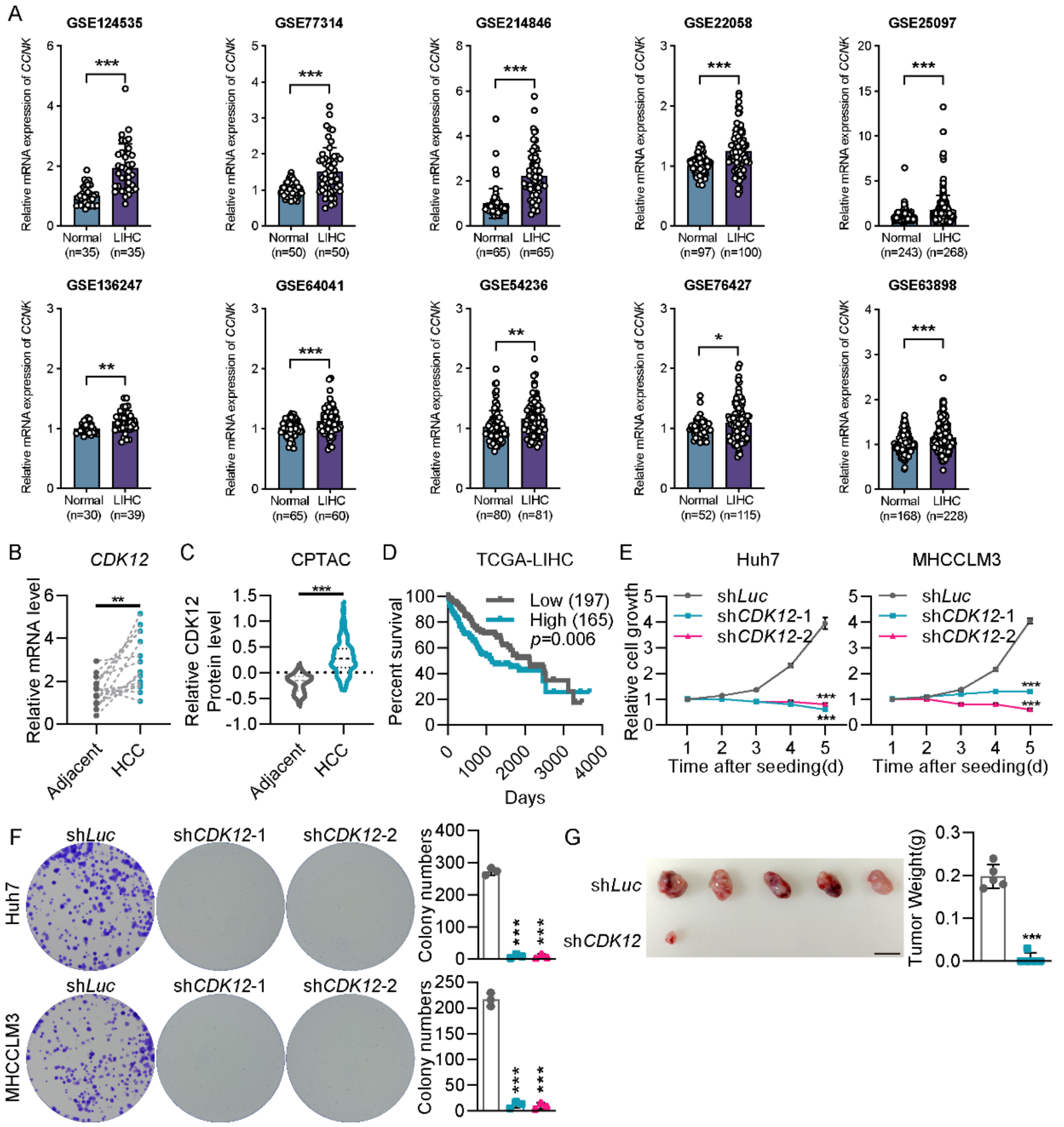

Fig. S1. CDK12/Cyclin K are upregulated in HCC associated with cell survival capability and patient clinical outcome.

(A) CCNK is elevated in HCC tissues derived from multiple GEO datasets. (B) Determination of CDK12 expression in 15 paired HCC and adjacent tissues (n=15). (C) The protein abundance of CDK12 is significantly elevated in HCC compared with adjacent non-tumor tissues derived from CPTAC dataset. (D) Survival analysis in patients with HCC stratified by CDK12 expression in TCGA datasets. (E) Evaluation of cell proliferation in Huh7 and MHCCLM3 cells upon CDK12 depletion by CCK8 assays. (F) The effect of cell viability in Huh7 (upper) and MHCCLM3 (below) cells upon CDK12 depletion. Quantification of the colony numbers were shown (right). (G) Xenograft growth in nude mice derived from Huh7 cells with or without CDK12 depletion (n=5). Quantification of xenografts weight were shown (right). Unpaired t test was used in A, B, C and G to determine statistical

significance. For F, *p* values were determined by one-way ANOVA. Data are presented as mean ± SD. *, **, *** means $p < 0.05$, $p < 0.01$, and $p < 0.001$.

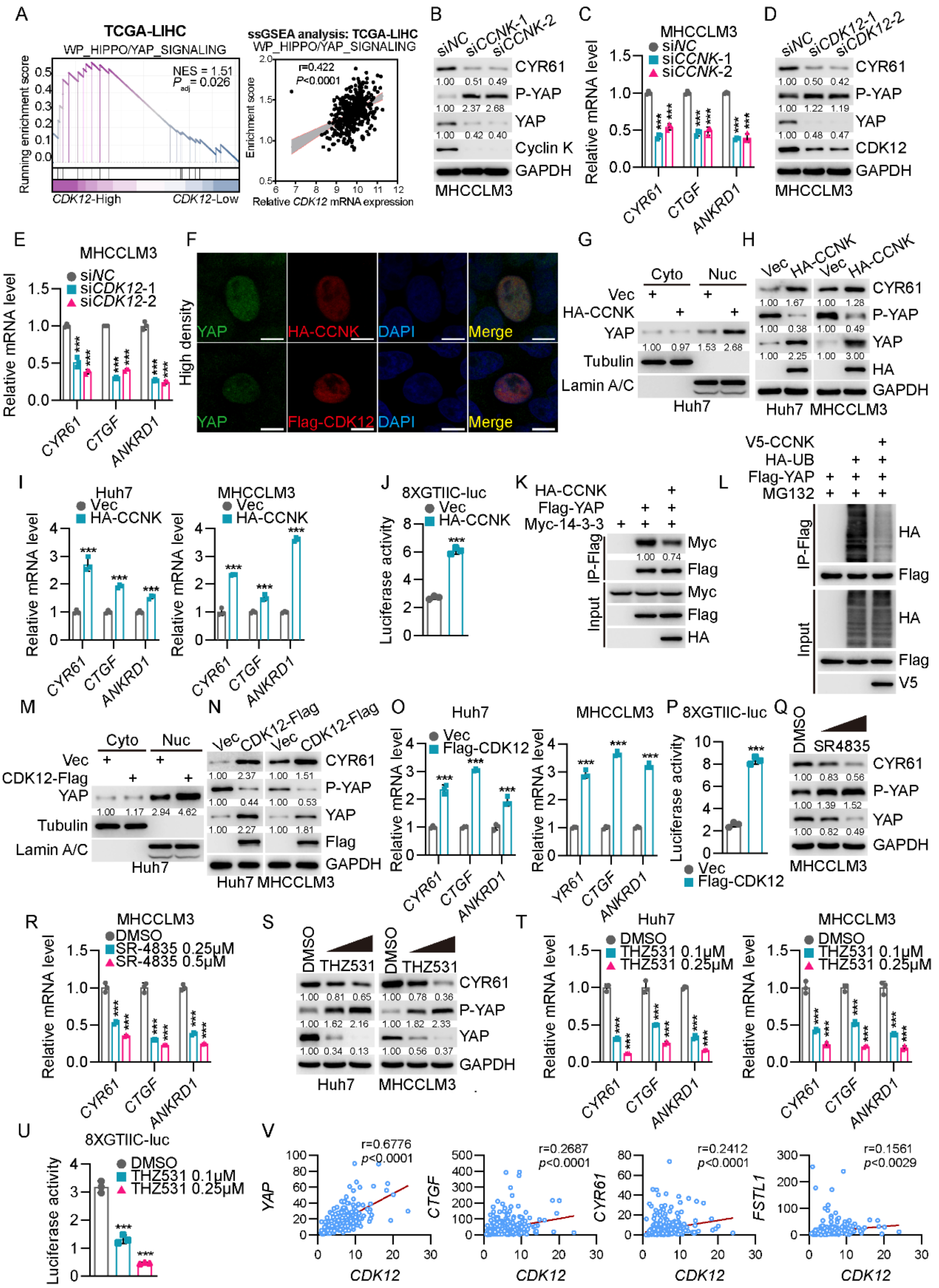

Fig. S2. CDK12/Cyclin K induced YAP activation in HCC.

(A) High CDK12 expression is associated with YAP signaling analyzed by GSEA and ssGSEA using

datasets derived from TCGA-LICH. (B, C) YAP signaling was attenuated in MHCCLM3 cells upon CCNK ablation. MHCCLM3 with or without CCNK depletion were subjected to immunoblotting (B) or qPCR analysis (C) as indicated. (D, E) CDK12 knockdown in MHCCLM3 cells suppresses YAP and its target expression. MHCCLM3 cells transfected with control and siRNA targeting CDK12 were subjected to immunoblotting (D) and qPCR analysis (E). (F) YAP subcellular distribution in Huh7 cells with high density upon CCNK (upper) or CDK12 (lower) expression. Scale bar, 10 μm. (G) YAP subcellular distribution was determined in Huh7 cells with or without CCNK overexpression. (H, I) Evaluation of the effect of CCNK overexpression on YAP signaling in HCC cells. Huh7 and LM3 cells with or without CCNK overexpression were subjected to immunoblotting (H) or qPCR analysis (I). (J) Analysis of 8×GTIIC-luc reporter activity in HEK-293T cells with or without CCNK overexpression. (K) Co-IP analysis of YAP/14-3-3 interaction in the presence of CCNK. HEK-293T cells transfected with Flag-YAP, Myc-14-3-3 with or without CCNK were subjected to Co-IP and immunoblotting. (L) Analysis of YAP ubiquitination with or without CCNK. HEK-293T cells transfected with Flag-YAP, HA-UB with or without CCNK were treated with MG132 and subjected to Co-IP and immunoblotting. (M) YAP subcellular distribution was determined in Huh7 cells with or without CDK12 overexpression. (N, O) Evaluation of YAP signaling in HCC cells upon CDK12 overexpression. Huh7 or MHCCLM3 with or without CDK12 overexpression were subjected to immunoblotting (N) and qPCR analysis (O). (P) Analysis of 8×GTIIC-luc reporter activity in HEK-293T cells with or without CDK12 overexpression. (Q, R) Evaluation of YAP activity in MHCCLM3 cells upon SR-4835 treatment (0.25 and 0.5 μM) for 8 h. MHCCLM3 with or without SR-4835 treatment were subjected to immunoblotting (Q) and qPCR analysis (R). (S, T) Evaluation of YAP activity in Huh7 cells upon THZ531 treatment (0.1 and 0.25 μM) for 8 h. Huh7 or MHCCLM3 with or without THZ531 treatment were subjected to immunoblotting (S) and qPCR analysis (T). (U) Analysis of 8×GTIIC-luc reporter activity in HEK-293T cells with or without THZ531 treatment (0.1 and 0.25 μM) for 8 h. (V) Correlation analysis of CDK12 expression with YAP, CTGF, CYR61 and FSTL1 using dataset from TCGA. the Pearson’s correlation test was used to analyze the link. For C, E, R, T, and U, *p* values were determined by one-way ANOVA. Unpaired t test was used in I, J, O, and P to determine statistical significance. Data are presented as mean ± SD. *, **, *** means $p < 0.05$, $p < 0.01$, and $p < 0.001$.

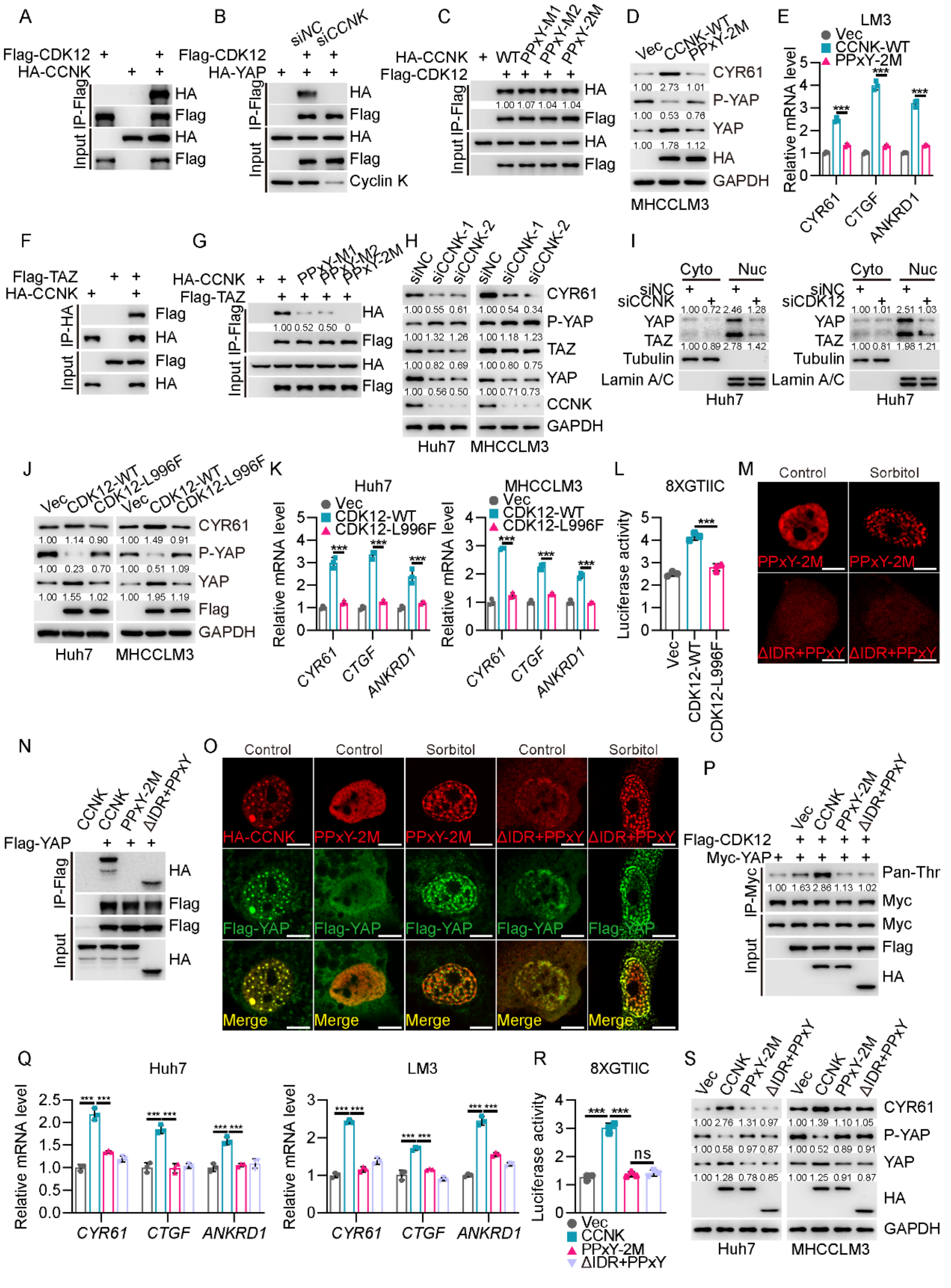

Fig. S3. The intact CDK12/Cyclin K complex is required to induce YAP activation.

(A) Validation of CDK12/Cyclin K interaction. (B) Evaluation of CDK12/YAP interaction in the absence of CCNK. HEK-293T cells transfected with Flag-CDK12, YAP with or without CCNK were subjected to Co-IP and immunoblotting. (C) The effect of Cyclin K mutant defective in YAP/Cyclin K binding on its association with CDK12. HEK293T cells cotransfected CDK12 with Vector, wild-type CCNK or

PPxY-2M were subjected to immunoprecipitation and immunoblotting. (D, E) YAP activation was attenuated in MHCCLM3 cells with Cyclin K defective in YAP/Cyclin K interaction. MHCCLM3 with Vector, wild-type CCNK or PPxY-2M were subjected to immunoblotting (D) and qPCR analysis (E). (F) Validation of TAZ/Cyclin K interaction. (G) Analysis of Cyclin K mutant defective in YAP/Cyclin K binding on its association with TAZ. (H) Analysis of YAP/TAZ signaling activity in HCC cells with CCNK depletion. (I) Determination of YAP subcellular localization in Huh7 cells upon CCNK or CDK12 knockdown. (J, K) YAP activation was attenuated in HCC cells with CDK12 defective in CDK12/Cyclin K interaction. Huh7 or MHCCLM3 with Vector, wild-type CDK12 or CDK12-L996F were subjected to immunoblotting (J) and qPCR analysis (K). (L) Analysis of 8×GTIIC-luc reporter activity in HEK-293T cells with Vector, wild-type CDK12 or CDK12-L996F. (M) Analysis of condensate formation in CCNK mutants in Huh7 cells with or without sorbitol treatment (0.2M, 20 min). Scale bar, 10 µm. (N) Evaluation of YAP binding to CCNK and its mutants. (O) Determination of YAP condensate formation and colocalization in Huh7 cells with or without sorbitol treatment (0.2M, 20 min). Scale bar, 10 µm. (P) Analysis of YAP T398 phosphorylation in HEK293T cells in the presence of CCNK, PPxY-2M and ΔIDR+PPXY. (Q) The mRNA levels of YAP target genes in Huh7 and MHCCLM3 cells upon CCNK, PPxY-2M and △IDR+PPXY expression. (R) Analysis of TEAD-responsive luciferase reporter activity in HEK293T cells upon CCNK, PPxY-2M and ΔIDR+PPXY transfection. (S) Evaluation of the effect of CCNK or its mutant overexpression on YAP signaling in HCC cells. For E, K, L, Q and R, *p* values were determined by one-way ANOVA. Data are presented as mean ± SD. *, **, *** means $p < 0.05$, $p < 0.01$, and $p < 0.001$.

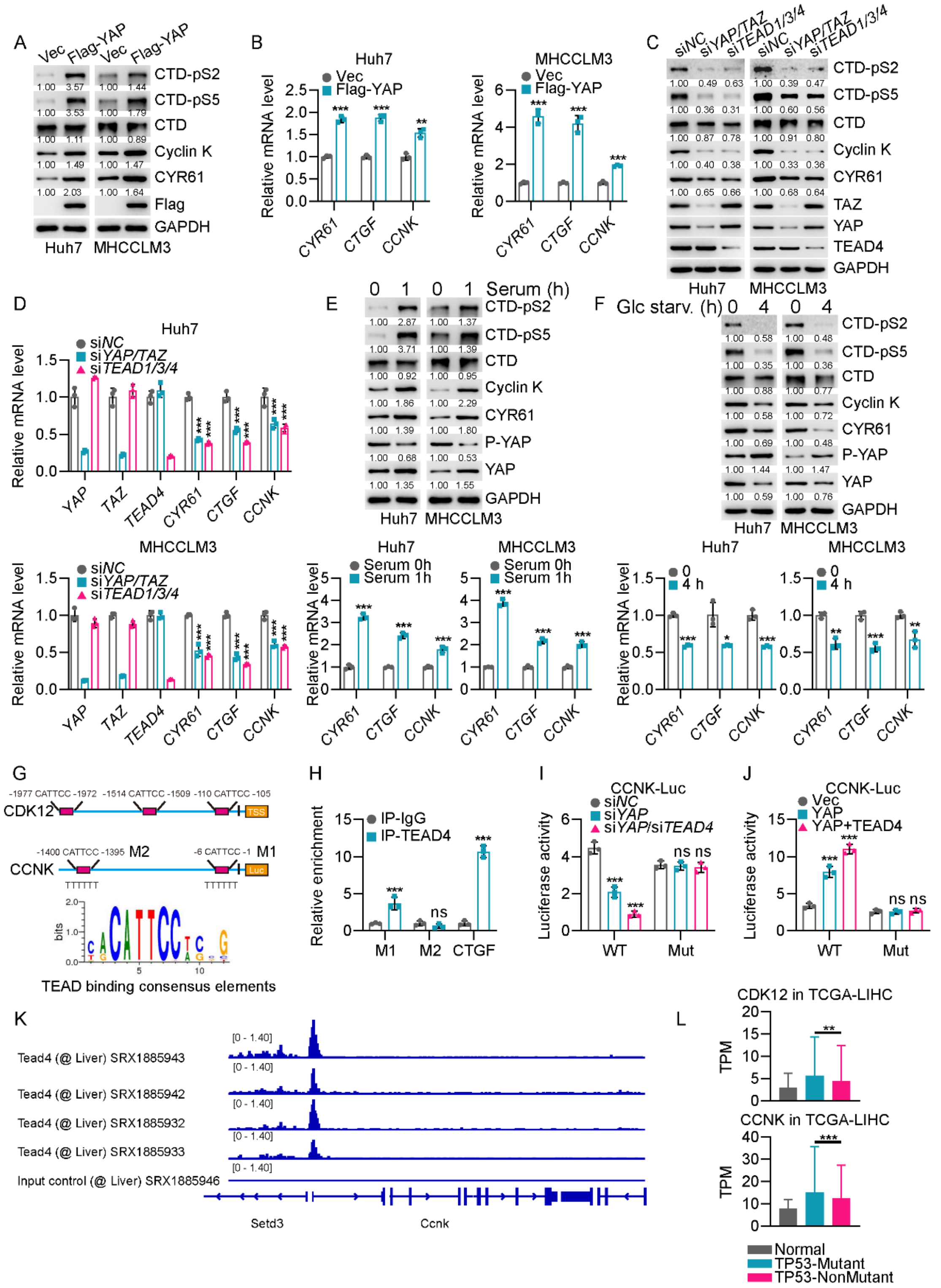

Fig. S4. CCNK is a bona fide target of YAP/TEAD4.

(A, B) CCNK expression was induced in HCC cells with YAP overexpression. Huh7 or MHCCLM3 with YAP overexpression were subjected to immunoblotting (A) and qPCR analysis (B). (C, D) CCNK expression was greatly reduced in HCC cells upon either YAP/TAZ or TEAD1/3/4 knockdown. Huh7 or MHCCLM3 with YAP/TAZ or TEAD1/3/4 knockdown were subjected to immunoblotting (C) and

qPCR analysis (D). (E) Evaluation of CCNK expression in HCC cells in response to serum stimulation. Huh7 or MHCCLM3 with glucose starvation for indicated times were subjected to immunoblotting (upper) and qPCR analysis (below). (F) Evaluation of CCNK expression in HCC cells in response to glucose starvation (Glc starv.). Huh7 or MHCCLM3 with glucose starvation for indicated times were subjected to immunoblotting (upper) and qPCR analysis (below). (G) schematic diagram of CDK12 and CCNK promoter region harboring TEAD4 binding motif. (H) Quantification of CCNK promoter fragment enrichment enriched by TEAD4. CTGF promoter fragment was used a positive control. (I) Analysis of CCNK-luc or CCNK-luc mutant reporter activity in HEK-293T cells with either YAP/TAZ or TEAD1/3/4 knockdown. (J) Analysis of CCNK-luc or CCNK-luc mutant reporter activity in HEK-293T cells with either YAP or TEAD4 overexpression. (K) IGV browser coverage tracks displaying ChIP-seq data on CCNK gene region generated in mouse liver by TEAD4. (L) Analysis of CDK12 and CCNK levels in TP53-mutant and non-mutant HCC. Unpaired t test was used in B, E, F and H to determine statistical significance. For D, I, J and L, *p* values were determined by one-way ANOVA. Data are presented as mean ± SD. *, **, *** means $p < 0.05$, $p < 0.01$, and $p < 0.001$.

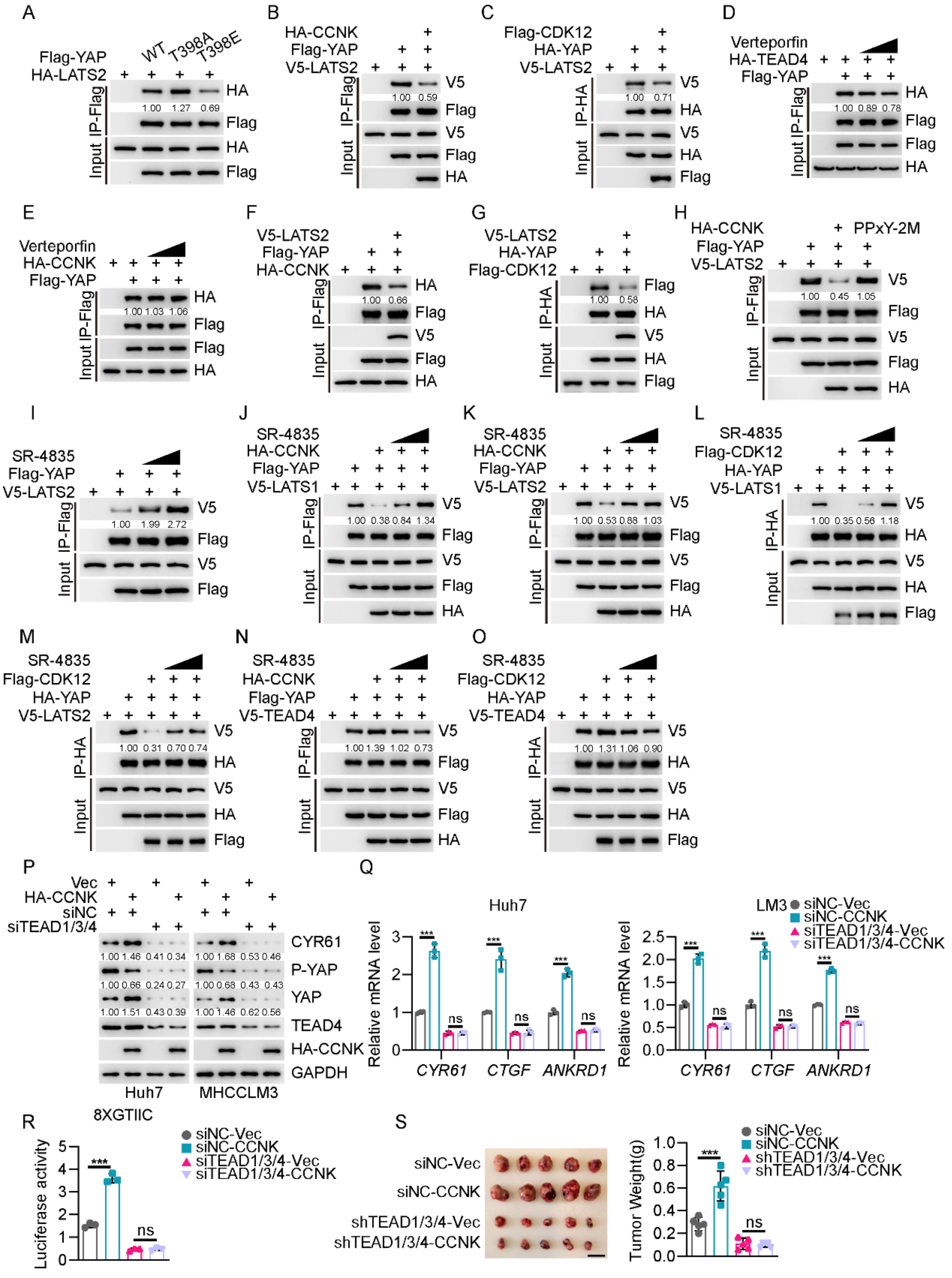

Fig. S5. CDK12/Cyclin K prevent LATS1/2-mediated YAP inactivation.

(A) YAP phosphorylation by CDK12/Cyclin K prevents its binding to LATS2 kinase. HEK-293T cells transfected with HA-LATS2 along with Flag-YAP, Flag-YAP-T398A and Flag-YAP-T398E were subjected to Co-IP and immunoblotting. (B) Cyclin K perturbs YAP/LATS2 interaction. HEK-293T cells transfected with V5-LATS2 and Flag-YAP with or without CCNK were subjected to Co-IP and

immunoblotting. (C) CDK12 perturbs YAP/LATS2 interaction. HEK-293T cells transfected with V5-LATS2 and HA-YAP with or without CDK12 were subjected to Co-IP and immunoblotting. (D, E) Analysis of verteporfin treatment (1, 2.5 μM) on YAP association with TEAD4 (D) and CCNK (E). (F, G) LATS2 inhibits YAP association with Cyclin K and CDK12. HEK-293T cells transfected with CCNK (F) or CDK12 (G) along with YAP with or without LATS2 were subjected to Co-IP and immunoblotting. (H) Cyclin K defective in YAP binding losses its ability to suppress LAST2/YAP interaction. (I) CCNK inhibitor SR-4835 impairs LAST2/YAP interaction. HEK-293T cells transfected with YAP and LATS2 were treated with SR-4835 (0.25 and 0.5 μM) for 8h for subsequent Co-IP and immunoblotting. (J, K) SR-4835 restores Cyclin K-mediated YAP disassociation with LATS1 (J) and LATS2 (K). (L, M) SR-4835 reverses CDK12-mediated YAP disassociation with LATS1 (L) and LATS2 (M). SR-4835 treatment (0.25 and 0.5 μM) for 8h was used. (N, O) SR-4835 interferes with YAP/TEAD4 binding caused by CDK12/Cyclin K. (P, Q) Evaluation of TEADs on CCNK-induced YAP activation or target gene expression in HCC cells. Huh7 or MHCCLM3 infected with vector or CCNK were transfected with or without siRNA against TEAD1/3/4, and analyzed by immunoblot or qPCR. (R) Analysis of TEAD-responsive luciferase reporter activity in HEK293T cells with indicated transfection. (S) Quantification of xenograft weight in indicated groups (n=5). Gross images of xenografts in nude mice from indicated Huh7 cells were showed. Scale bar, 10 mm. For Q, R and S, p values were determined by one-way ANOVA. Data are presented as mean ± SD. *, **, *** means $p < 0.05$, $p < 0.01$, and $p < 0.001$.

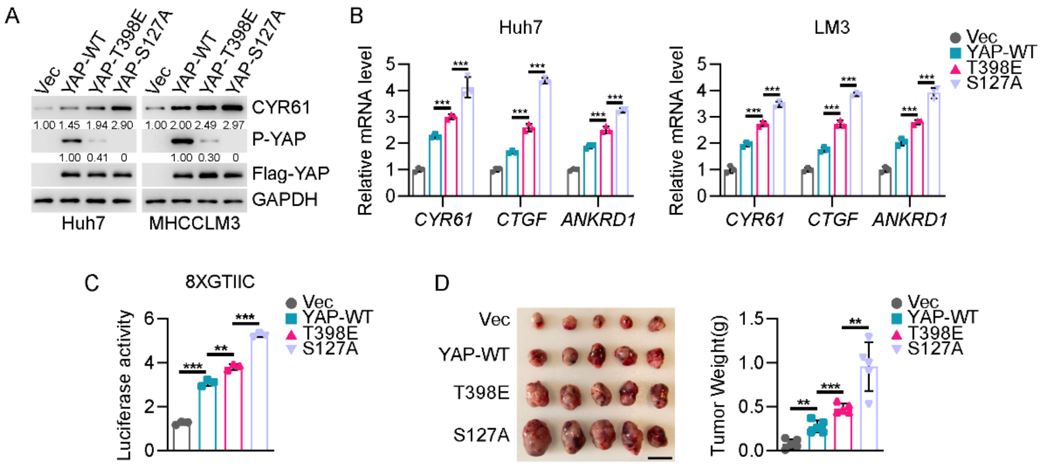

Fig. S6. T398 phosphorylation is critical for YAP oncogenic activity.

(A, B) Immunoblot (A) and qPCR (B) analysis of YAP activation and target genes expression in HCC cells with YAP or indicated mutants. (C) Analysis of TEAD-responsive luciferase reporter activity in HEK293T cells with YAP or indicated mutant transfection. (D) Quantification of xenograft weight in indicated groups (n=5). Gross images of xenografts in nude mice from indicated Huh7 cells were

showed. Scale bar, 10 mm. For B, C and D, p values were determined by one-way ANOVA. Data are presented as mean ± SD. *, **, *** means p < 0.05, p < 0.01, and p < 0.001.

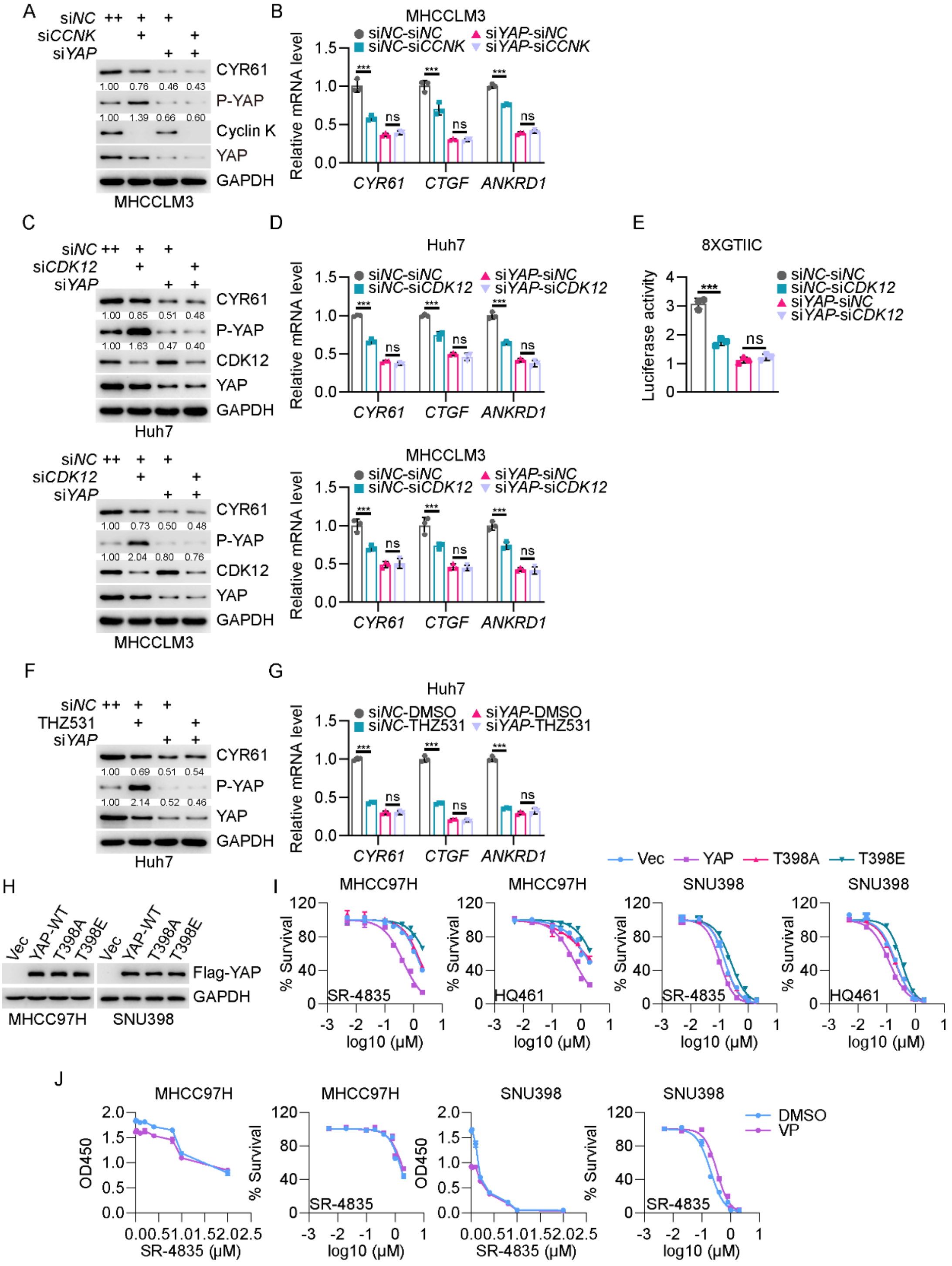

Fig. S7. YAP mediates downstream effect of CDK12/Cyclin K.

(A, B) YAP ablation abolishes CCNK-mediated Hippo signaling inhibition. Control or CCNK-depleted

MHCCLM3 cells were transfected with or without siRNA targeting YAP and subjected to immunoblotting (A) and qPCR analysis (B). (C-E) YAP ablation abolishes CDK12-mediated Hippo signaling inhibition. Control or CDK12-depleted Huh7 and MHCCLM3 cells were transfected with or without siRNA targeting YAP and subjected to immunoblotting (C), qPCR analysis (D) and reporter activity analysis (E). (F, G) YAP knockdown overcomes CDK12 inhibition-induced YAP suppression and target gene repression. Control or YAP-depleted Huh7 cells were treated with or without THZ531 and subjected to immunoblotting (F) and qPCR analysis (G). (H) Immunoblot verification of YAP and indicated mutant expression. (I) Quantification of HCC cell sensitivity (IC50) to Cyclin K molecular glue degraders in indicated cell lines with YAP and indicated mutant expression. (J) The effect of verteporfin treatment (1μM) on the HCC cell sensitivity to SR4835. MHCC97H and SNU398 were treated with or without verteporfin treatment in the presence of serial doses of SR4835 for 48h. The growth curve and normalized response to SR4835 were showed. For B, D, E and G, p values were determined by one-way ANOVA. Data are presented as mean ± SD. *, **, *** means $p < 0.05$, $p < 0.01$, and $p < 0.001$.

Supplementary Table 1: Potential Interacting partners of Cyclin K identified by mass spectrometry.

Supplementary Table 2: YAP peptide characterization prior to CDK12 kinase reaction.

Supplementary Table 3: Phosphopeptide mapping of YAP from in vitro CDK12 kinase assay.
